# Antimicrobial activity and possible mechanisms of juglone against *Escherichia coli*, *Staphylococcus aureus*, and *Salmonella pullorum*

**DOI:** 10.1186/s12866-025-04354-0

**Published:** 2025-10-17

**Authors:** Lei Wang, Mingxin Qiu, Xuanyue Li, Mengjian Liu, Luyu Li, Yong Chen

**Affiliations:** Research Center for Biofeed and Animal Gut Health, College of Animal Science, Xinjiang Agricultural University, No.311 Nongda East Road, Urumqi, Xinjiang 830052 China

**Keywords:** Juglone, Antimicrobial mechanism, Transcriptomic sequencing, Cell structure, Energy metabolism, Pathogenic bacteria

## Abstract

**Background:**

*Escherichia coli*, *Staphylococcus aureus*, and *Salmonella pullorum* are significant pathogens that threaten livestock and poultry health. Although antibiotics and synthetic antimicrobial agents can combat these pathogens, antibiotic resistance remains a major concern. Recent decades have seen growing interest in antibiotic alternatives. Juglone, a natural naphthoquinone compound from Juglandaceae plant, exhibits strong antimicrobial activity against *S. aureus*. However, its antimicrobial mechanism is not yet fully understood. This study investigated the antimicrobial mechanism of juglone from the perspectives of cell biology, cell morphology, and transcriptomics.

**Results:**

Juglone had potent antimicrobial effects against *E. coli*, *S. aureus*, and *S. pullorum*. The minimum inhibitory concentration (MIC) of juglone against all three bacterial strains was 15.6 µg/mL. Treatment with juglone decreased bacterial metabolic activity, reduced the intracellular DNA and RNA fluorescence intensity, resulted in the leakage of intracellular alkaline phosphatase (AKP) and ions, and caused a decline in ATP content and ATPase activity. Scanning electron microscopy (SEM) revealed significant membrane damage in each of the three bacterial species following juglone treatment. Transcriptomic sequencing and Gene Ontology (GO) enrichment analysis of *S. pullorum* revealed that juglone treatment resulted in a significant upregulation of GO terms related to translation, while those terms associated with transport, localization, and membrane functions were significantly downregulated. Kyoto Encyclopedia of Genes and Genomes (KEGG) pathway analysis showed that the pathways related to oxidative phosphorylation and the citrate cycle were significantly upregulated, whereas those pathways related to ABC transporters and quorum sensing were significantly downregulated.

**Conclusion:**

These findings suggest that juglone compromises the permeability and integrity of the cell envelope in *E. coli*, *S. aureus*, and *S. pullorum*, resulting in cytoplasmic leakage and metabolic impairment. Additionally, juglone alters the gene expression of transporters, interferes with the energy metabolism, protein synthesis and transport, quorum sensing, and biofilm formation of *S. pullorum*, thereby exerting antimicrobial effects.

**Supplementary Information:**

The online version contains supplementary material available at 10.1186/s12866-025-04354-0.

## Introduction

Pathogenic bacteria are a significant cause of disease in livestock and poultry, and these pathogens and their toxins cause high morbidity and mortality rates among these animals. For example, *Staphylococcus aureus* is known to induce mastitis in dairy cows, resulting in a substantial decrease in milk yield and a decline in milk quality [1]. Pathogenic *Escherichia coli* is capable of readily inducing colibacillosis in poultry, with mortality rates among chicks reported to be between 5% and 20% [2]. Following *Salmonella pullorum* infection, the pathogen causes severe damage to chick intestinal tracts and microbiota, subsequently disseminating to host tissues and organs and resulting in systemic lesions [3]. Antibiotics play crucial and positive roles in preventing and treating these diseases. However, the emergence of antibiotic-resistant pathogens due to misuse and overuse of antibiotics poses an increasing threat to both human and animal health. In 2019, approximately 4.95 million deaths globally were associated with bacterial resistance [4]. It has been estimated that if the misuse of antibiotics is not effectively controlled, nearly 10 million people could die from antibiotic-resistant bacterial infections by 2050 [5]. Antibiotic resistance can be transferred among humans, animals, and environmental microorganisms. Compared with that of antibiotics used in humans, antibiotic resistance in animals requires more attention. The addition of antibiotics to animal feed as growth promoters is prevalent and exhibits a remarkable growth trend in low- and middle-income countries. Antibiotic resistance has led to increased mortality rates in livestock and poultry, decreased production performance, and economic losses [6]. Reducing or even banning the use of antibiotics as growth promoters is an effective strategy for controlling antibiotic resistance in microorganisms. Currently, alternatives to antibiotics used in feed include antimicrobial peptides, bacteriophages, probiotics, nanoparticles, and phytobiotics, with phytobiotics being considered the most promising [7, 8].

Phytobiotics, also known as phytochemicals or phytogenics, are substances extracted from plants that possess antimicrobial, anti-inflammatory, and immune-enhancing properties. They primarily include bioactive compounds such as phenolics, alkaloids, and phytosterols and are used in animal husbandry to improve animal health and production performance. For example, clove essential oil has been shown to enhance growth performance, improve immune-antioxidant status, and modify the jejunal morphology and microbiota composition of heat-stressed broilers [9]. Eugenol, the principal component of clove oil, can improve broiler production performance, mitigate the negative effects of *E. coli* O78 infection, and control the colonization and dissemination of *Salmonella* spp [10, 11]. Rosemary extract not only exhibits antimicrobial activity against several pathogens but also enhances the production performance and meat quality of ducks through its antioxidant and anti-inflammatory effects [12, 13]. Walnut (*Juglans regia* L.) green husk extract has significant antimicrobial activity against *S. aureus*, *Bacillus subtilis*, and *E. coli*, which is associated with the presence of phenolics, flavonoids, and terpenoids [14]. Juglone (5-hydroxy-1,4-naphthoquinone), the primary active constituent in walnut green husk, exhibits broad-spectrum antimicrobial activity against various bacterial strains, including methicillin-resistant *S. aureus* (MRSA), *Listeria monocytogenes*, and *Bacillus cereus* [15–17]. Although juglone demonstrates moderate antibacterial activity compared to conventional antibiotics such as penicillin G and amphotericin B, it exhibits a lower propensity for inducing drug resistance. This advantage stems from its mechanism of action, wherein juglone triggers the generation of reactive oxygen species (ROS) within bacterial cells, leading to systemic cellular damage [18, 19]. Additionally, juglone exhibits a range of bioactivities, including notable antioxidant [20], antiviral [21], and antifungal properties [19], and presents considerable potential for the development of novel antimicrobial agents for livestock and poultry.

After treatment with juglone, the expression of proteins in *S. aureus* related to protein synthesis, oxidative damage, DNA replication and transcription, and the stress response was suppressed [22, 23]. Previous studies have employed transcriptome sequencing to investigate the antibacterial mechanism of walnut green husk extract against *E. coli* [14], providing valuable insights into the inhibitory effects of juglone on *E. coli*. *S. pullorum* is an important pathogen responsible for livestock and poultry diseases. However, whether juglone exhibits similar antibacterial activity against *S. pullorum* and how it affects gene expression at the transcriptional level remain unexplored. Therefore, the objectives of this study are: first, to evaluate the antibacterial effects of juglone against *S. aureus*, *E. coli*, and *S. pullorum* at the cellular level; and second, using *S. pullorum* as a model organism, to elucidate its antibacterial mechanism at the molecular level through transcriptome sequencing. The findings will provide a scientific basis for the potential application of juglone in the prevention and treatment of animal diseases.

## Materials and methods

### Strains and reagents

*E. coli* CVCC1382, *S. aureus* CVCC2257 and *S. pullorum* CVCC525 were purchased from the National Center for Veterinary Culture Collection (Beijing, China). Juglone (purity > 97%, CAS: 481-39-0) was obtained from Sigma‒Aldrich (St. Louis, MO, USA). Alkaline phosphatase (AKP), ATP, and ATPase assay kits were procured from Nanjing Jiancheng Bioengineering Institute (Nanjing, China). Flavomycin (purity ≥ 99%) was purchased from North China Pharmaceutical Corporation (Shijiazhuang, China). RNA extraction kits were obtained from TianGen Biotech (Beijing, China). 4’,6-Diamidino-2-phenylindole (DAPI) staining solution, iodonitrotetrazolium chloride (INT), and all other reagents used in the experiments were of analytical grade and were purchased from Sangon Biotech (Shanghai, China).

### Minimum inhibitory concentration (MIC) of juglone

The MIC of juglone was determined via the Oxford cup method [24] to assess its antimicrobial activity against *E. coli*, *S. aureus*, and *S. pullorum*. Briefly, juglone was dissolved in anhydrous ethanol to prepare a stock solution of 1.0 mg/mL, which was then serially diluted twofold to prepare working solutions. A 0.1 mL aliquot of fresh bacterial suspension (1 × 10^8^ colony-forming units (CFU)/mL) was added to 15 mL of sterile Luria–Bertani (LB) agar media at 50 °C, mixed, and poured into Petri dishes. After the media solidified, Oxford cups (ø 8 mm) were placed on the agar, and each cup was filled with 200 µL of diluted juglone-ethanol solution. The plates were incubated at 4 °C for 12 h in the dark to allow juglone to diffuse into the agar, followed by incubation at 37 °C for 12 h. The diameters of the inhibition zones (excluding the diameter of the Oxford cups) were measured. Each treatment was performed in triplicate, and three measurements were taken per replicate. The MIC of juglone was defined as the concentration at which complete inhibition of bacterial growth occurred. Anhydrous ethanol and flavomycin (50 µg/mL) served as negative and positive controls, respectively.

### Effects of juglone on the growth of bacteria

Juglone solutions were added to 50 mL of liquid LB media to achieve final concentrations of 0, 0.5, 1, and 2 × MIC. A 50 µL aliquot of fresh bacterial inoculum was then introduced into each culture. The cultures were incubated in a water bath shaker (ZWYR-200D, Zhicheng Analytical Instrument Manufacturing Co., Ltd., Shanghai, China) at 37 °C and 170 rpm for 24 h. Every 2 h, 0.2 mL samples were withdrawn, and the absorbance at 600 nm (A_600nm_) was measured using a microplate reader (Infinite M200, TECAN, Switzerland).

### Ion leakage, intracellular ATP content and ATPase activity

Ion leakage, the intracellular ATP content and ATPase activity were measured according to previous methods with slight modifications [14]. In brief, the bacteria were grown in liquid LB media to a concentration of 1 × 10^8^ CFU/mL. Juglone solutions were then added, and the cultures were incubated at 37 °C with shaking at 170 rpm for 12 h. Samples of 10.0 mL were collected at 0, 2, 4, 8, and 12 h and then centrifuged at 4 °C and 3200 × *g* for 10 min. The resulting supernatants were diluted tenfold with double-distilled water (ddH_2_O), and the conductivity was measured using a conductivity meter (FE38, Mettler-Toledo, Shanghai, China) to assess the extent of ion leakage. The cell pellets were washed three times, resuspended in 10.0 mL of saline, and subjected to ultrasonic disruption. The samples were then centrifuged at 4 °C (3200 × *g* for 10 min for ATP and 1300 × *g* for 5 min for ATPase activity). After centrifugation, the supernatants were collected for subsequent analysis. Intracellular ATP levels were quantified using an ATP assay kit, while the enzymatic activities of Na^+^/K^+^-ATPase, Mg^2+^-ATPase, and Ca^2+^-ATPase were determined using specific ATPase assay kits. Protein concentration in the supernatants was determined via the Bradford method [25] using bovine serum albumin as the standard.

### Extracellular AKP activity

The bacteria were cultured in liquid LB media until they reached the logarithmic growth phase. The cultures were harvested and centrifuged at 4 °C and 1800 × *g* for 10 min. The supernatant was discarded, and the bacterial pellet was resuspended in sterile saline. An additional 30.0 mL of liquid LB media was added to achieve a final bacterial concentration of 1 × 10^8^ CFU/mL. Juglone solutions were then added, and the cultures were incubated at 37 °C with shaking at 170 rpm for 12 h. Samples of 1.0 mL were collected at 0, 2, 4, 8, and 12 h postincubation and then centrifuged at 4 °C and 18,000 × *g* for 5 min. AKP activity in the supernatants was measured using a commercial AKP assay kit according to the manufacturer’s instructions.

### Cellular metabolic activity

Bacteria were cultured to a concentration of 1 × 10^8^ CFU/mL. Juglone was then added, and the mixture was incubated at 37 °C for 2 h. The samples were then centrifuged at 3200 × *g* for 15 min, and the resulting cell pellets were collected, washed three times, and resuspended to a concentration of 1 × 10^8^ CFU/mL in saline. INT solution was added to a final concentration of 1 mmol/L, and the samples were incubated at 37 °C for 30 min. The absorbance was measured at 630 nm (A_630_) to evaluate cellular metabolic activity.

### Nucleic acid fluorescence intensity

The intracellular nucleic acid fluorescence intensity was measured according to a previous method with slight modifications [23]. In brief, a 0.8 mL aliquot of bacterial culture treated with juglone for 12 h was immediately mixed with 2.4 mL DAPI staining solution diluted fourfold with ddH_2_O. The mixture was then incubated in a shaking water bath at 170 rpm for 10 min. The fluorescence intensities of the DNA and RNA in the bacterial cells were recorded using a spectrofluorometer (RF-5301PC, Shimadzu, Kyoto, Japan), with an emission wavelength of 454 nm and excitation wavelengths of 364 nm and 400 nm.

### Cell morphology

A 5.0 mL aliquot of bacterial culture, treated with juglone for 6 h, was centrifuged at 1800 × *g* for 10 min. The supernatant was discarded, and the pellet was resuspended in 1.0 mL of formalin-aceto-alcohol fixative (FFA) at 4 °C for 12 h. Following fixation, the samples were centrifuged again at 1800 × *g* for 10 min. The cell pellets were dehydrated in graded ethanol solutions (50–100%), and each step lasted 15 min. The pellets were then resuspended in 1.0 mL of tert-butanol and stored at 4 °C until the tert-butanol was completely solidified. The freeze-dried bacterial samples were cathodically sprayed with platinum, and the morphology of the bacteria was subsequently observed using a field emission scanning electron microscope (Quanta FEG250, FEI, La Vergne, TN, USA).

### Transcriptome sequencing of S. pullorum

*S. pullorum* was cultured to a concentration of 1 × 10^8^ CFU/mL, and juglone was added to achieve a final concentration of 1 × MIC. The mixture was then incubated at 37 °C with shaking at 170 rpm for 6 h. The treated group (T group) and the control group (C group) each consisted of six replicates. From each replicate, a 5.0 mL sample was collected and centrifuged, and the bacterial pellets were washed with sterile 0.1 M phosphate-buffered saline (PBS, pH 7.4). Total RNA from *S. pullorum* was extracted using a commercial kit (TianGen Biotech, Beijing, China), and RNA integrity was assessed using an Agilent 2100 Bioanalyzer (Agilent Technologies, Palo Alto, CA, USA). The enriched total RNA was fragmented and used as a template to synthesize complementary DNA (cDNA). The purified cDNA underwent end repair, A-tailing, and ligation of the sequencing adapter. cDNA fragments ranging from 370 to 420 bp in length were selected for library construction after PCR amplification, and the sequencing library was sequenced on a NovaSeq 6000 platform (Illumina, San Diego, CA, USA).

After quality control analysis of the raw reads, clean data were obtained. Sequence alignment was performed via Bowtie2 software (v 2.3.4), and the *Salmonella enterica* subsp. enterica serovar pullorum str. ATCC 9120 genome was used as the reference (https://www.ncbi.nlm.nih.gov/datasets/genome/GCF_000330485.2/). Gene expression levels were quantified via RSEM software (v1.3.1) and normalized to fragments per kilobase of transcript per million mapped reads (FPKMs). Gene Ontology (GO) term enrichment analysis was conducted via the GOseq R package (v 1.54.0), and Kyoto Encyclopedia of Genes and Genomes (KEGG) pathway analysis was performed via KOBAS software (v 3.0). Gene set enrichment analysis (GSEA) and heatmap generation were conducted at https://magic.novogene.com/.

### Statistical analysis

The data were analyzed via SPSS software (v 19.0) (SPSS Inc., Chicago, IL, USA), and the results are expressed as the means ± standard deviations (means ± SDs) of three independent replicates. One-way analysis of variance (ANOVA) followed by Duncan’s multiple range test was employed to assess differences among the treatment groups. *p* < 0.05 was considered statistically significant. Analysis of differentially expressed genes (DEGs) from the transcriptomic sequencing data was performed via the DESeq2 R package (v 1.20.0). Adjusted P values were calculated via the Benjamini and Hochberg (BH) procedure, and thresholds for differential gene expression were set at an adjusted *p* ≤ 0.05 and |log2(fold change) | ≥ 1.

## Results

### Antibacterial activity and MIC

The inhibition zone diameters of the juglone agonists *E. coli*, *S. aureus* and *S. pullorum* are presented in Table 1. Juglone exhibited favorable inhibitory effects on these bacterial strains. However, at concentrations lower than 15.6 µg/mL, juglone did not show antibacterial activity against any of the three bacteria. Consequently, the MIC of juglone for all three bacterial strains was determined to be 15.6 µg/mL.

**Table 1:** Zone diameters (mm) of the juglone agonists *E. coli*, *S. aureus* and *S. pullorum*

Bacteria	Juglone (µg/mL)								Ethanol	Flavomycin (50 µg/mL)
	500	250	125	62.5	31.25	15.60	7.80	3.90		
*E. coli*	10.67 ± 0.58	7.83 ± 0.76	7.50 ± 0.50	6.33 ± 0.58	3.33 ± 0.29	2.67 ± 0.29	0	0	0	4.11 ± 0.54
*S. aureus*	10.83 ± 0.76	9.67 ± 0.29	8.00 ± 1.00	6.33 ± 1.04	3.33 ± 0.29	2.00 ± 0.00	0	0	0	12.61 ± 0.63
*S. pullorum*	10.50 ± 0.50	9.83 ± 1.04	8.33 ± 0.58	6.83 ± 1.04	4.33 ± 0.29	3.33 ± 0.29	0	0	0	8.78 ± 0.51

Values are expressed as the means ± standard deviations (SDs) of three independent replicates

### Growth curve

**Fig. 1:**
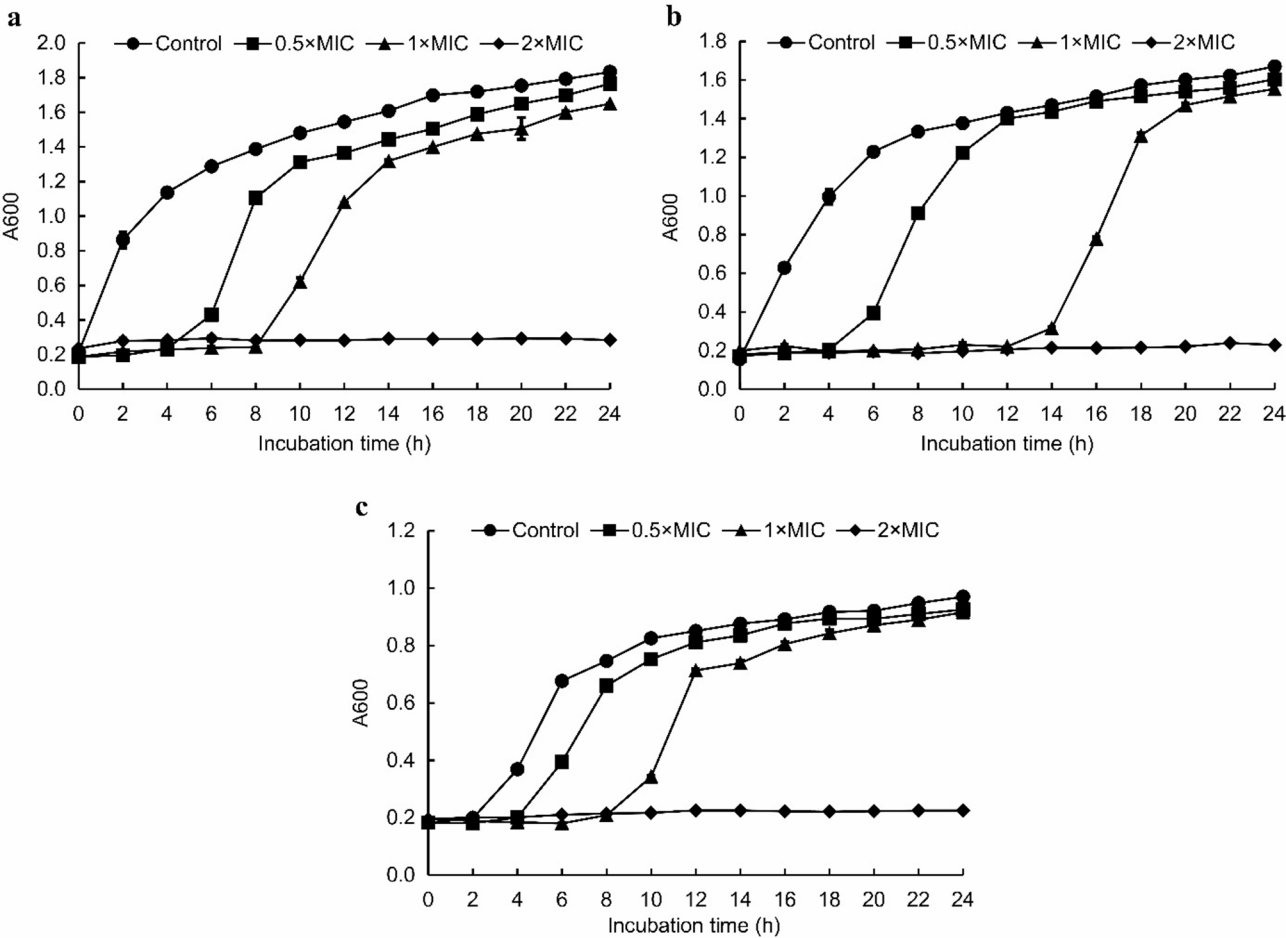
Effects of juglone on the growth of *E. coli* (**a**), *S. aureus* (**b**), and *S. pullorum* (**c**). The error bars indicate the standard deviations (SDs) of three independent replicates

The effects of different concentrations of juglone on the growth of *E. coli*, *S. aureus*, and *S. pullorum* are shown in Fig. 1. With 0.5 × MIC juglone, the bacteria entered the logarithmic growth phase after 4 h of incubation. When exposed to 1 × MIC juglone, *E. coli* (Fig. 1a) and *S. pullorum* (Fig. 1c) entered the logarithmic growth phase after 8 h of incubation, whereas *S. aureus* (Fig. 1b) entered this phase after 12 h of incubation. All these bacterial strains were completely inhibited by 2 × MIC juglone.

### Ion leakage

**Fig. 2:**
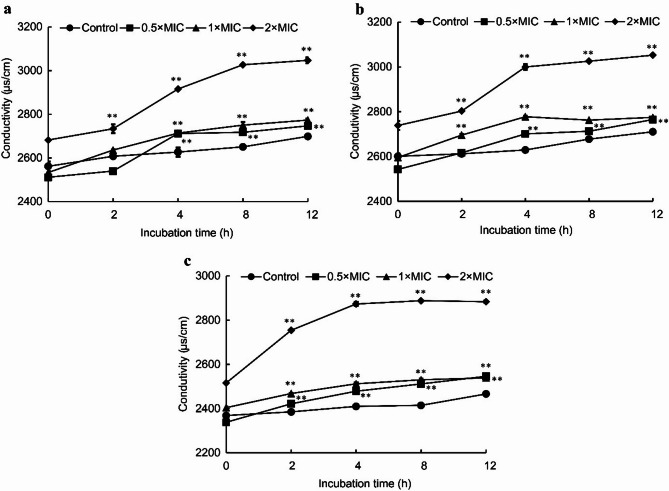
Effects of juglone on the conductivity of the diluted culture media for *E. coli* (**a**), *S. aureus* (**b**), and *S. pullorum* (**c**). The error bars indicate the standard deviations (SDs) of three independent replicates. *p < 0.05 and **p < 0.01 compared with the control group

Leakage of intracellular ions into the culture media leads to an increase in the conductivity of the media. The effects of juglone on the conductivity of the diluted culture media are illustrated in Fig. 2. For *E. coli*, exposure to 2 × MIC juglone for 2 h significantly increased the conductivity of the media (*p* < 0.01), and similar increases were observed at 4 h in the presence of 0.5 × MIC and 1 × MIC juglone (*p* < 0.01) (Fig. 2a). For *S. aureus*, the media conductivity was significantly greater at 2 h in the presence of 1 × MIC and 2 × MIC juglone (*p* < 0.01) and at 4 h in the presence of 0.5 × MIC juglone (*p* < 0.01) (Fig. 2b). For *S. pullorum*, the media conductivity significantly increased after 2 h of exposure to 0.5 × MIC, 1 × MIC, and 2 × MIC juglone (*p* < 0.01) (Fig. 2c).

### Intracellular ATP content

**Fig. 3:**
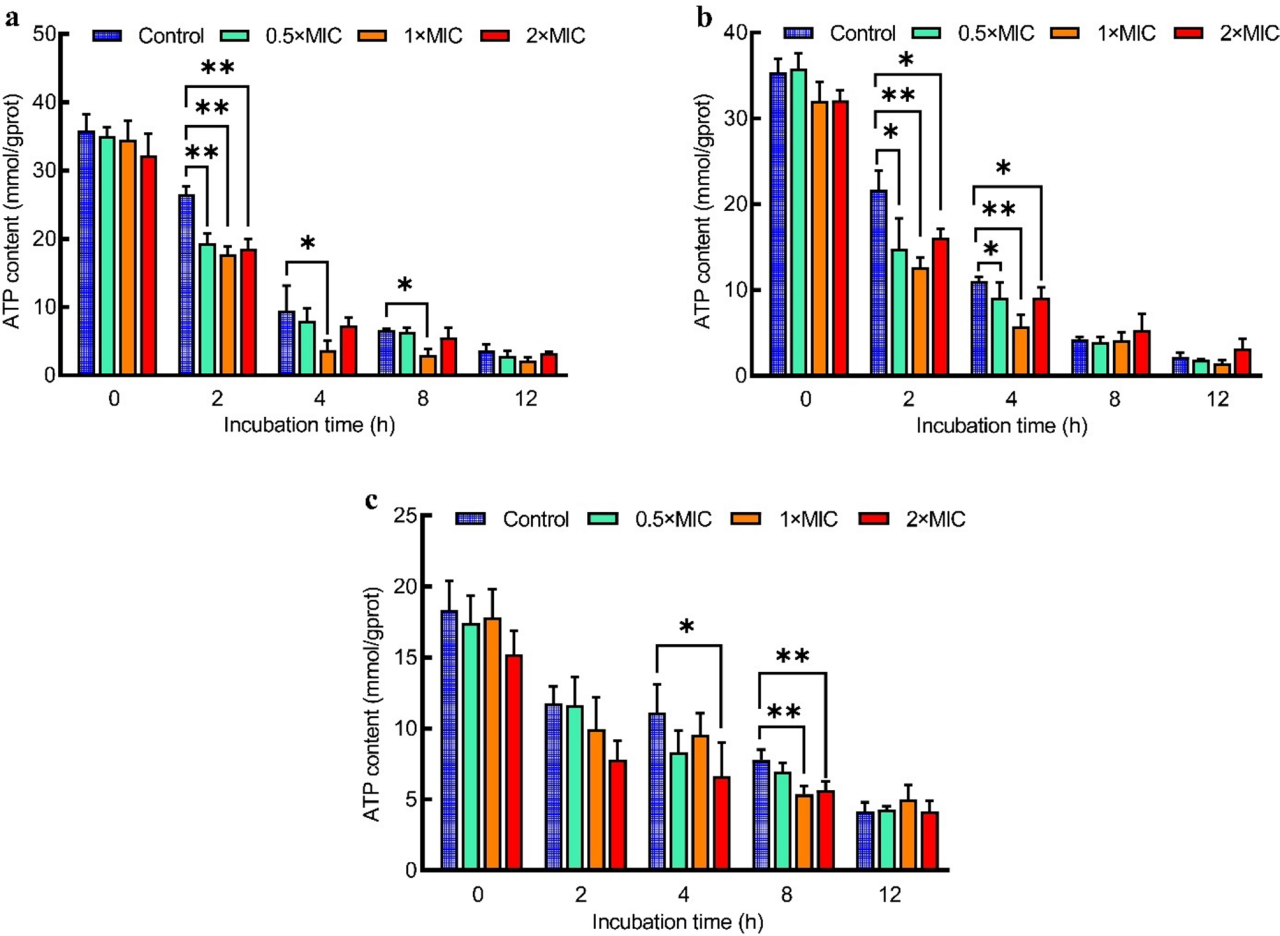
Effects of juglone on the intracellular ATP content of *E. coli* (**a**), *S. aureus* (**b**), and *S. pullorum* (**c**). The error bars indicate the standard deviations (SDs) of three independent replicates. *p < 0.05 and **p < 0.01 compared with the control group

The effects of juglone on the intracellular *E. coli*, *S. aureus*, and *S. pullorum* ATP contents are shown in Fig. 3. *E. coli* ATP concentrations were significantly reduced after 2 h of juglone treatment at 1 × MIC (*p* < 0.01), and this reduction persisted until the end of 8 h (*p* < 0.01) (Fig. 3a). *S. aureus* ATP concentrations were significantly lower than those in the control group at both 2 h and 4 h following juglone treatment (*p* < 0.05 and *p* < 0.01) (Fig. 3b). *S. pullorum* ATP levels were significantly lower than those in the control group at 4 h and 8 h after exposure to 2 × MIC juglone (*p* < 0.05 and *p* < 0.01, respectively) and significantly lower at 8 h following treatment with 1 × MIC juglone (*p* < 0.01).

### Intracellular ATPase activity

**Fig. 4:**
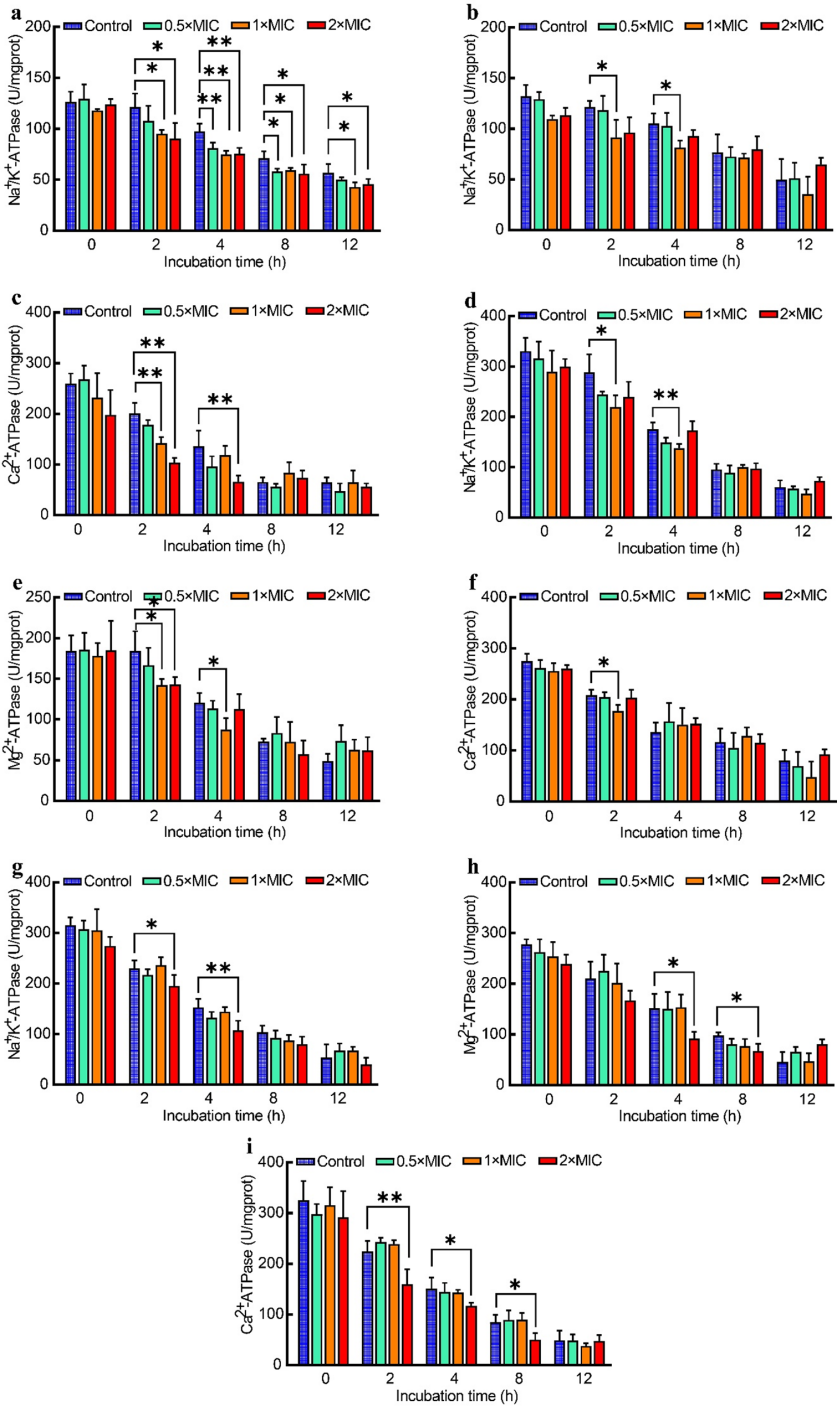
Effects of juglone on *E. coli* (**a**, **b**, **c**), *S. aureus* (**d**, **e**, **f**), and *S. pullorum* (**g**, **h**, **i**) intracellular ATPase activity. The error bars indicate the standard deviations (SDs) of three independent replicates. * *p* < 0.05 and ** *p* < 0.01 compared with the control group

The effects of juglone on ATPase activity in *E. coli*, *S. aureus*, and *S. pullorum* are illustrated in Fig. 4. In *E. coli*, Na^+^/K^+^-ATPase activity significantly decreased after 2 h of treatment with 1 × MIC and 2 × MIC juglone (*p* < 0.05), and it was significantly reduced by treatment with 0.5 × MIC juglone at 4 h and 8 h (*p* < 0.05) (Fig. 4a). Mg^2+^-ATPase activity was significantly lower than that of the control group at 2 h and 4 h following treatment with 1 × MIC juglone (*p* < 0.05) (Fig. 4b). Ca^2+^-ATPase activity was significantly reduced at 2 h posttreatment with 1 × MIC juglone (*p* < 0.01) and at 2 h and 4 h posttreatment with 2 × MIC juglone (*p* < 0.01) (Fig. 4c). In *S. aureus*, the activities of Na^+^/K^+^-ATPase (Fig. 4d) and Mg^2+^-ATPase (Fig. 4e) significantly decreased at 2 h and 4 h following treatment with 1 × MIC juglone (*p* < 0.05 and *p* < 0.01, respectively), and Ca^2+^-ATPase activity (Fig. 4f) was significantly lower at 2 h posttreatment (*p* < 0.05) than in the control group. Compared with the control, treatment with 2 × MIC juglone led to a significant reduction in Na^+^/K^+^-ATPase activity (g) at 2 h and 4 h (Fig. 4g), Mg^2+^-ATPase activity at 4 h and 8 h (Fig. 4h), and Ca^2+^-ATPase activity at 2 h, 4 h, and 8 h in *S. pullorum* (Fig. 4i) (*p* < 0.05 and *p* < 0.01, respectively).

### Extracellular AKP activity

**Fig. 5:**
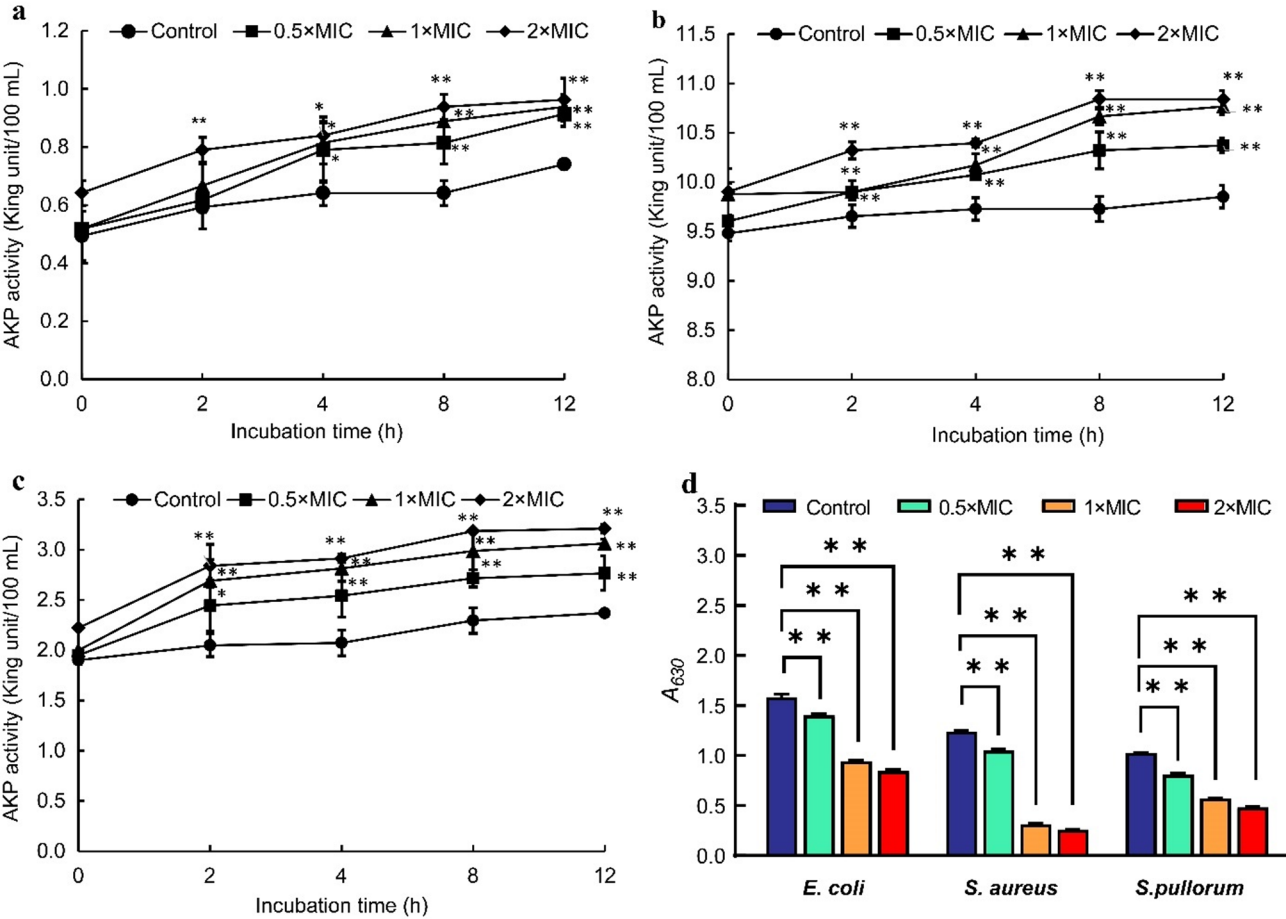
Effects of juglone on the extracellular AKP activity of *E. coli* (**a**), *S. aureus* (**b**), and *S. pullorum* (**c**), as well as on metabolic activity (**d**). The error bars indicate the standard deviations (SDs) of three independent replicates. *P < 0.05 and ** *p* < 0.01 compared with the control group

The effects of juglone on *E. coli*, *S. aureus*, and *S. pullorum* extracellular AKP activity are shown in Fig. 5. Compared with that in the control group, AKP activity in the *E. coli* culture media significantly increased after 2 h of treatment with 2 × MIC juglone (*p* < 0.05) and after 4 h of treatment with 0.5 × MIC and 1 × MIC juglone (*p* < 0.05) (Fig. 5a). For *S. aureus* (Fig. 5b) and *S. pullorum* (Fig. 5c), AKP activity was also significantly improved even after 2 h of treatment with 0.5 × MIC juglone (*p* < 0.05 and *p* < 0.01).

### Cellular metabolic activity

The effects of juglone on the metabolic activity of *E. coli*, *S. aureus*, and *S. pullorum* are shown in Fig. 5d. Following treatment with 0.5 × MIC, 1 × MIC, or 2 × MIC juglone, the cellular metabolic activity of these bacteria was significantly lower than that of the control bacteria (*p* < 0.01).

### Nucleic acid fluorescence intensity

**Fig. 6:**
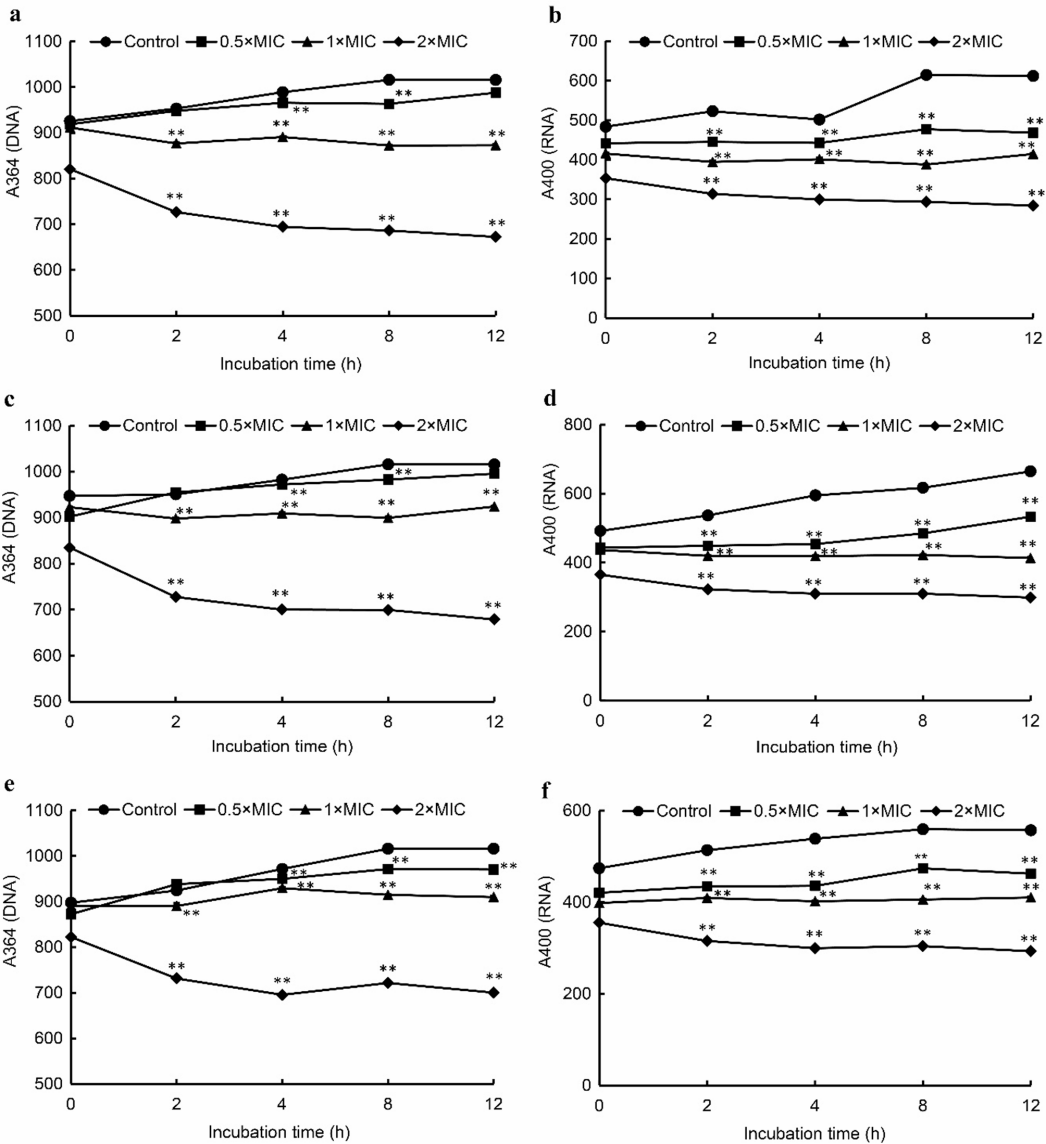
Effects of juglone on the DNA and RNA fluorescence intensities from *E. coli* (**a**, **b**), *S. aureus* (**c**, **d**), and *S. pullorum* (**e**, **f**). The error bars indicate the standard deviations (SDs) of three independent replicates. *p < 0.05 and **p < 0.01 compared with the control group

The effects of juglone on the DNA and RNA fluorescence intensity of *E. coli*, *S. aureus*, and *S. pullorum* are shown in Fig. 6. The fluorescence intensities of DNA (A_364_) and RNA (A_400_) from the control group, including *E. coli* (Fig. 6a, b), *S. aureus* (Fig. 6c, d), and *S. pullorum* (Fig. 6e, f), increased with increasing incubation time. Following treatment with juglone, the DNA and RNA fluorescence intensities decreased. Notably, after treatment with 1 × MIC or 2 × MIC juglone, the DNA and RNA fluorescence intensities significantly decreased with increasing incubation time (*p* < 0.01).

### Cell morphology

**Fig. 7:**
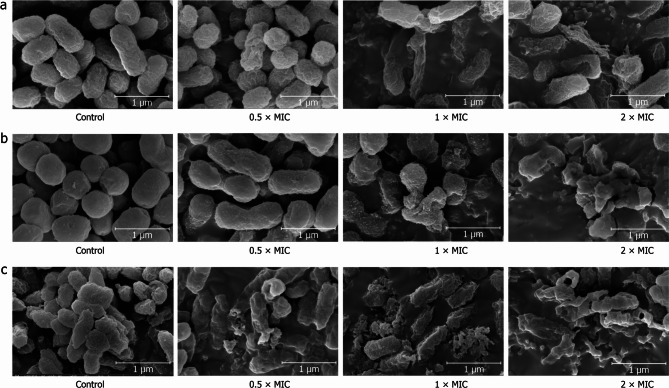
Effects of juglone extract on the morphology of *E. coli* (**a**), *S. aureus* (**b**), and *S. pullorum* (**c**). Bacteria in the experimental groups treated with 0.5, 1 or 2 × MIC juglone for 6 h. Images were obtained using a field emission scanning electron microscope equipped with Everhardt–Thornley detectors (ETD) at an accelerating voltage of 5.00 kV, a magnification of 30,000× and a working distance of 11.1 mm

Scanning electron microscopy images of *E. coli*, *S. aureus*, and *S. pullorum* are presented in Fig. 7. After treatment with 0.5 × MIC juglone, the cell surfaces of *E. coli* (Fig. 7a), *S. aureus* (Fig. 7b), and *S. pullorum* (Fig. 7c) exhibited dehydration and shrinkage. Following exposure to 1 × MIC and 2 × MIC juglone, the bacterial cells ruptured and even completely disintegrated.

### Transcriptome sequencing of S. pullorum

#### Data quality

After sequencing and quality control analysis, an average of 14.91 million clean reads per sample were obtained, with ≥ 2.0 G clean bases, a sequencing error rate ≤ 0.02%, a Q20 > 98.5%, and a Q30 > 94.5%. Using Bowtie2 software for alignment, an average of 98.78% of the reads were successfully mapped to the reference genome (ranging from 98.67 to 98.90%). Of these, 95.13% (ranging from 92.40 to 97.42%) were mapped to unique locations, whereas 3.65% (ranging from 1.33 to 6.37%) were mapped to multiple locations (see Table S1).

#### Analysis of DEGs

**Fig. 8:**
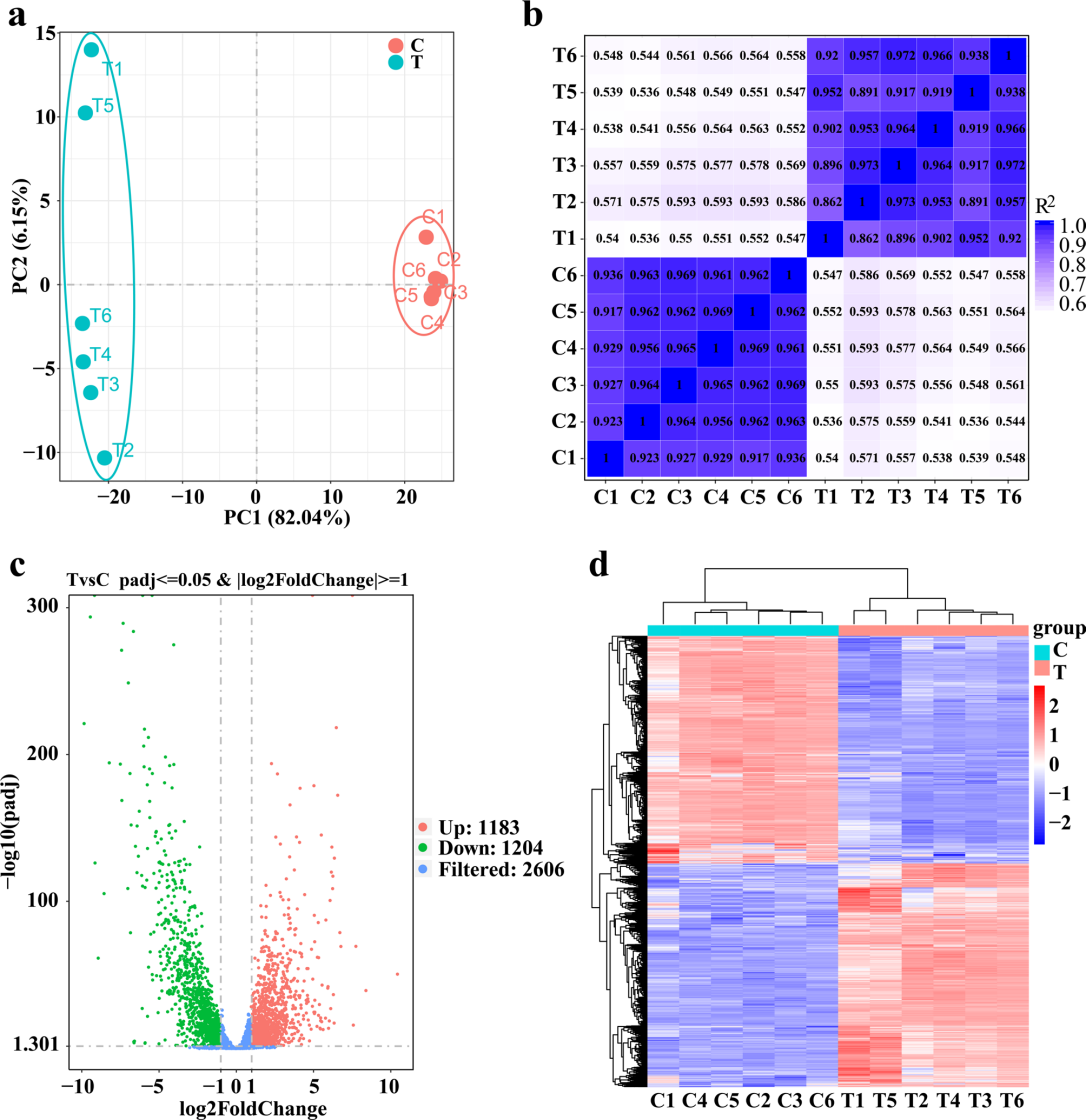
Principal component analysis (PCA) (**a**), Pearson correlation coefficients (**b**), volcano diagram (**c**), and heatmap (**d**) of differentially expressed genes (DEGs). (**a)** PCA of the gene expression levels of *S. pullorum* treated with juglone. The *x*-axis represents the first principal component (PC1), and the *y*-axis represents the second principal component (PC2). The distance between data points reflects their similarity, with points positioned more closely together indicating greater similarity. (**b)** Heatmap of the Pearson correlation coefficients (R^2^) between samples. The correlation coefficients within and between groups were calculated on the basis of the FPKM values for all genes expressed in each sample. A deeper blue color indicates a stronger correlation. (**c)** Volcano plot of DEGs after juglone treatment. The criteria for significant differential expression were *p*adj < 0.05 and |log2 (fold change) | >1. Red plots represent upregulated genes, green plots represent downregulated genes, and blue plots represent filtered genes. (**d)** Heatmap of all the DEGs. The *x*-axis represents the samples from the control and juglone-treated groups, and the *y*-axis represents different genes; red indicates genes exhibiting increased expression, and blue indicates genes exhibiting decreased expression. C, control; T, treatment with 1 × MIC juglone

Principal component analysis (PCA), Pearson correlation coefficient (R^2^), volcano diagrams, and heatmaps of the DEGs between the control and treatment groups are shown in Fig. 8. The results of PCA indicated that gene expression levels were more similar within the same group of replicate samples, while there were significant differences between different treatment groups, which were grouped into distinct clusters. PC1 and PC2 explained 82.04% and 6.15% of the total variance, respectively (Fig. 8a). Correlation analysis of gene expression levels revealed that the Pearson correlation coefficient within the control group exceeded 0.91, that within the experimental group exceeded 0.86, and that between the control and experimental groups was less than 0.60 (Fig. 8b). According to the criteria *p*adj ≤ 0.05 and |log2 (fold change) | ≥ 1, a total of 2387 DEGs were identified between the control and juglone-treated groups. Among these genes, 1183 genes presented upregulated expression, and 1204 genes showed downregulated expression (Fig. 8c). Clustering analysis of the DEGs revealed a clear segregation between the control and experimental groups (Fig. 8d).

#### GO terms and KEGG pathway analysis

**Fig. 9:**
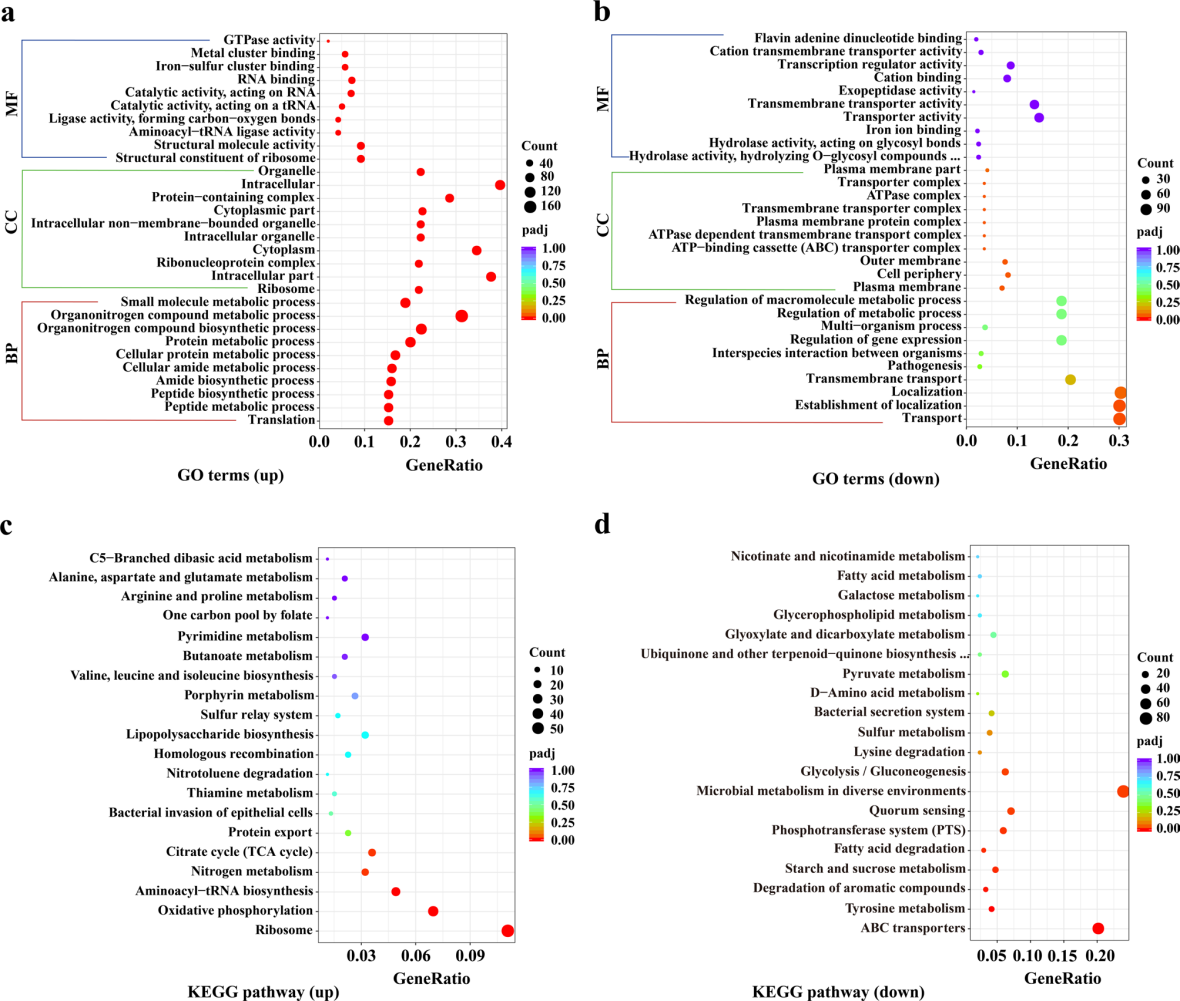
Scatter plot of differentially expressed genes (DEGs) according to Gene Ontology (GO) and Kyoto Encyclopedia of Genes and Genomes (KEGG) enrichment analyses. (**a)** The top 30 upregulated GO terms. (**b)** The top 30 downregulated GO terms. DEGs enriched in biological processes (BPs), cellular components (CCs), and molecular functions (MFs). (**c)** The top 20 upregulated KEGG pathways. (**d)** The top 20 downregulated KEGG pathways. The dot size represents the number of enriched genes

GO analysis revealed that the DEGs were enriched in a total of 477 GO terms, including 273 terms related to biological processes (BPs), 34 terms related to cellular components (CCs), and 170 terms related to molecular functions (MFs). The top 10 up- and downregulated GO terms in terms of BPs, CCs and MFs are illustrated in Fig. 9a and b. Among the enriched BP-related terms, a total of 31 terms were significantly enriched, including several related to protein biosynthesis, such as translation, peptide biosynthetic processes, tRNA aminoacylation, ribosome biogenesis, cation transmembrane transport, and drug metabolic processes (*p*adj < 0.05). Conversely, transport and establishment of localization were significantly downregulated (adjusted *P* < 0.05). In the CC category, a total of 13 terms, including ribosome, ribonucleoprotein complex, and intracellular organelle, were significantly upregulated, whereas 9 terms, including the plasma membrane and ATP-binding cassette (ABC) transporter complex (*p*adj < 0.05), were significantly downregulated. In the MF category, 14 terms were significantly upregulated, including structural constituents of ribosomes, structural molecule activity, and aminoacyl-tRNA ligase activity (*p*adj < 0.05), and no significantly downregulated terms were identified.

KEGG enrichment analysis revealed that DEGs were significantly enriched in 85 pathways. The top 20 up- and downregulated KEGG pathways are shown in Fig. 9c and d. Among these, pathways related to ribosomes, oxidative phosphorylation, aminoacyl-tRNA biosynthesis, nitrogen metabolism, and the citrate cycle were significantly upregulated (*p*adj < 0.05). Conversely, pathways related to ABC transporters, tyrosine metabolism, aromatic compound degradation, starch and sucrose metabolism, fatty acid degradation, the phosphotransferase system, quorum sensing, microbial metabolism in diverse environments, and glycolysis/gluconeogenesis were significantly downregulated (*p*adj < 0.05).

#### GSEA

##### Cell wall and cell membrane

**Fig. 10:**
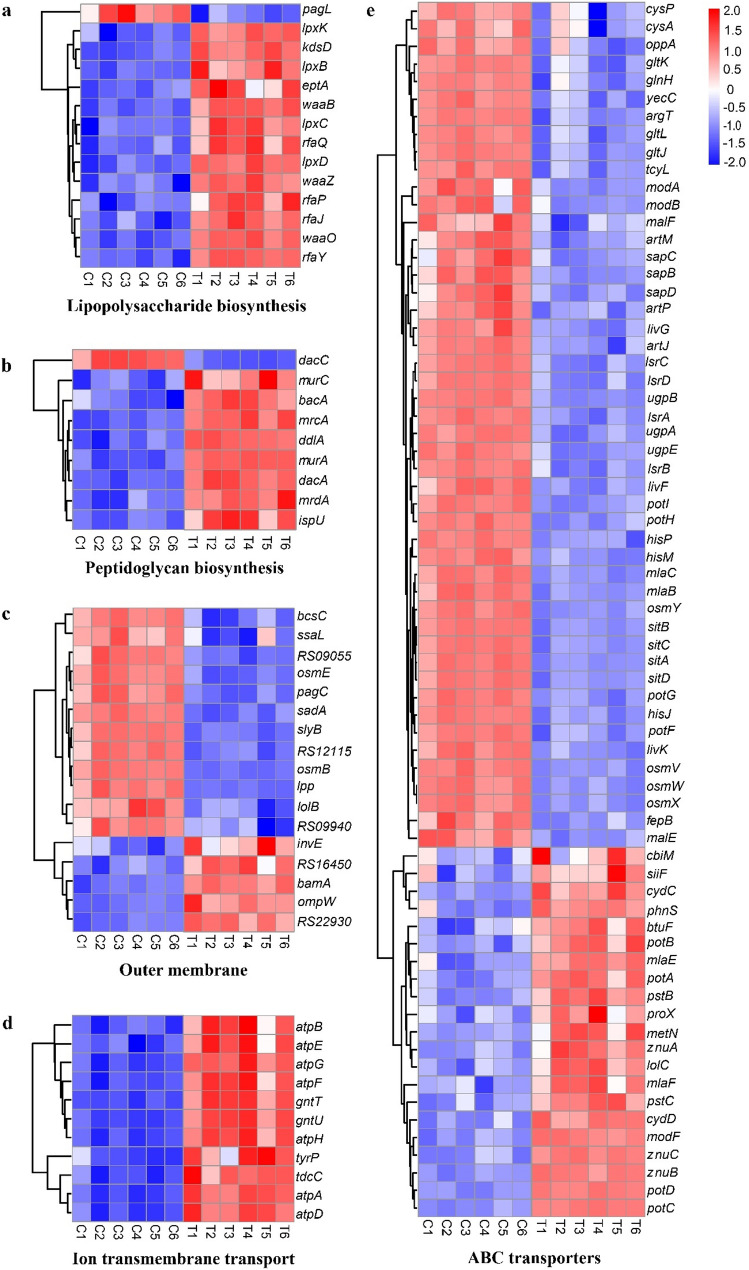
Heatmap of differentially expressed genes (DEGs) related to the cell wall and cell membrane, ranked by gene set enrichment analysis (GSEA). (**a)** Lipopolysaccharide biosynthesis. (**b)** Peptidoglycan biosynthesis. (**c)** Outer membrane. (**d)** Ion transmembrane transport. (**e)** ATP-binding cassette (ABC) transporters. Red indicates upregulated genes, whereas blue indicates downregulated genes

After treatment with 1 × MIC juglone, the expression of 13 out of 14 DEGs related to lipopolysaccharide (LPS) biosynthesis in *S. pullorum* was upregulated at the transcriptional level. These genes included those encoding LPS glycosyltransferases (*waaB*, *waaO*, *waaZ*, *rfaJ*, *rfaQ*, *rfaP*, and *rfaY*), a sulfatase (*eptA*), and glycolipid synthases (*lpxB*, *lpxC*, *lpxD*, and *lpxK*) (Fig. 10a).

Among the 9 DEGs related to peptidoglycan (PG) biosynthesis, 8 showed upregulated expression, including genes encoding carboxypeptidase (*dacA*), dipeptidase (*ddlA*), peptidoglycan glycosyltransferases (*mrcA*, *mrdA*), N-acetylglucosamine synthetase (*murA*, *murC*), and phosphatases (*ispU*, *bacA*) (Fig. 10b).

All 11 DEGs involved in ion transport, including genes encoding ATP synthase (*atp*) and gluconate transporters (*gnt*), were upregulated (Fig. 10c). Several genes encoding outer membrane lipoproteins (*lolB*, *osmB*, *osmE*, *pagC*, RS09940, RS12115, and *slyB*) were downregulated, whereas genes related to outer membrane protein repair (*bamA*, *ompW*, and RS16450) were upregulated (Fig. 10d).

Among the 69 genes associated with ABC transporters whose expression significantly changed, 21 were upregulated, and 48 were downregulated. The upregulated genes included those encoding transporters for glutathione/L-cysteine (*cydC*, *cycD*), polyamines (*potA*, *potB*, *potC*, and *potD*), phospholipids (*mlaE*, *mlaF*, and *phnS*), zinc (*znuA*, *znuB*, and *znuC*), and phosphate (*pstB*, *pstC*). Among the genes with downregulated expression, 24 were associated with amino acid or peptide transport, and others related to sulfate/thiosulfate (*cysA*, *cysP*), maltose (*malE*, *malF*), iron/manganese ions (*sitA*, *sitB*, *sitC*, *sitD*, and *fepB*), putrescine (*potF*, *potG*, *potH*, and *potI*), 3-phosphoglycerate (*ugpA*, *ugpB*, and *ugpE*), and autoinducer 2 (*lsrA*, *lsrB*, *lsrC*, and *lsrD*) were also downregulated (Fig. 10e).

##### Energy metabolism

**Fig. 11:**
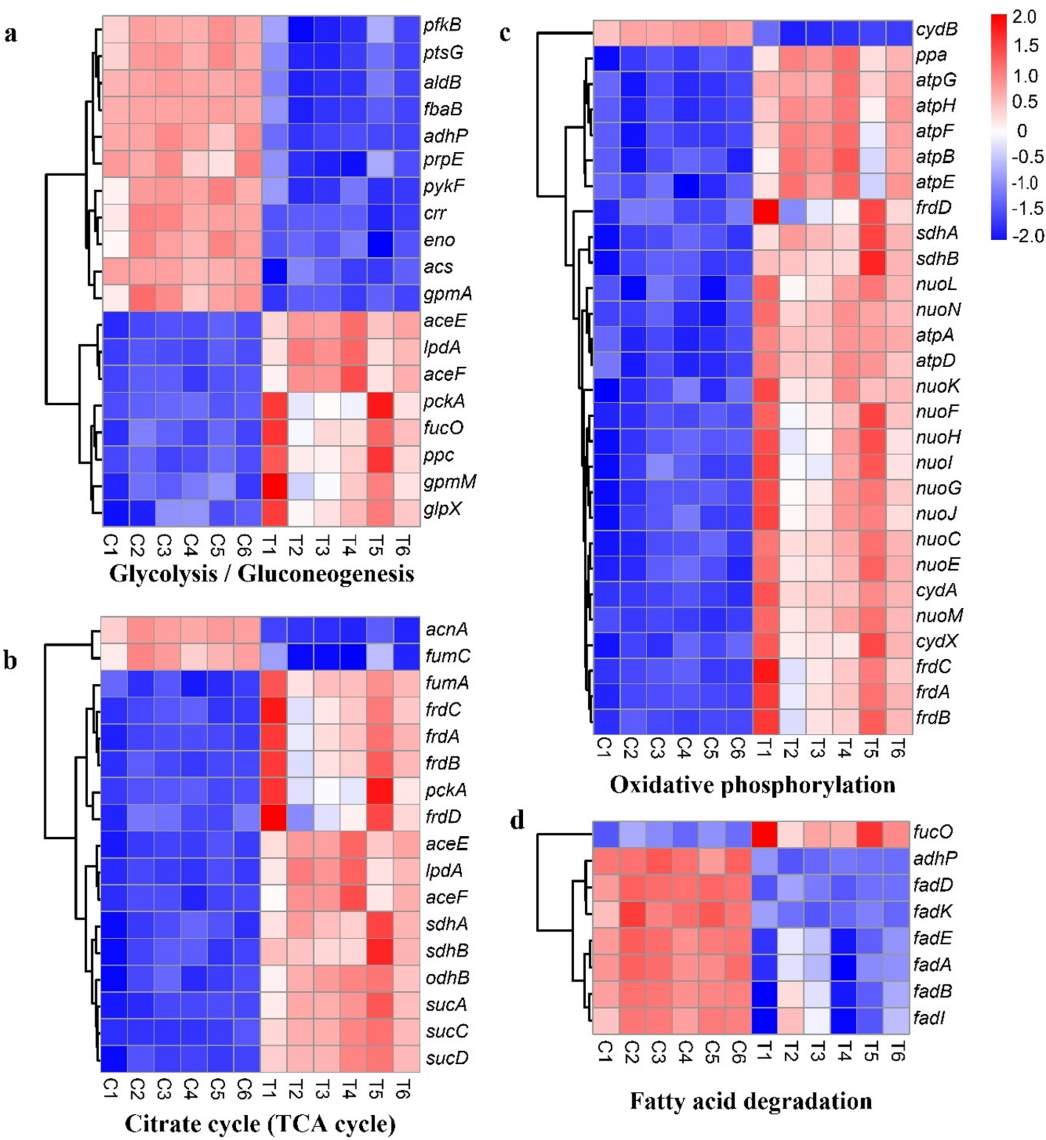
Heatmap of differentially expressed genes (DEGs) related to energy metabolism, ranked by gene set enrichment analysis (GSEA). (**a)** DEGs related to glycolysis/gluconeogenesis. (**b)** The citrate cycle (TCA cycle). (**c)** Oxidative phosphorylation. and (**d)** Fatty acid degradation

A total of 19 DEGs associated with glycolysis and gluconeogenesis were identified; 11 genes showed downregulated expression and 8 genes showed upregulated expression (Fig. 11a). The key enzymes in the glycolysis pathway include hexokinase, 6-phosphofructokinase-1 (pfkB), and pyruvate kinase (pykF). The expression levels of the hexokinase-encoding gene were unaffected by juglone treatment, whereas the expression levels of the *pfkB* and *pykF* genes were significantly downregulated. The key enzymes involved in gluconeogenesis include phosphoenolpyruvate carboxylase (ppc), phosphoenolpyruvate carboxykinase (pckA), fructose-1,6-bisphosphatase-1 (glpX), and glucose-6-phosphatase. Following juglone treatment, the expression levels of the *ppc*, *pckA*, and *glpX* genes were significantly increased (Fig. 11a), indicating that juglone treatment accelerated gluconeogenesis.

In the tricarboxylic acid (TCA) cycle, 17 DEGs were observed, with 15 genes showing upregulated expression and only 2 genes showing downregulated expression (Fig. 11b). Key enzyme-encoding genes in the TCA cycle, such as genes encoding the 2-oxoglutarate dehydrogenase complex (*sucA*, *odhB*, *lpdA*), succinate-CoA ligase (*sucD*), and succinate dehydrogenase (*sdhA*, *sdhB*, *frdA*, *frdB*, *frdC*, and *frdD*), all showed significantly upregulated expression.

Among the DEGs related to oxidative phosphorylation, 28 out of 29 genes showed upregulated expression, and only the *cydB* gene showed downregulated expression (Fig. 11c). These upregulated genes included 11 genes encoding NADH-quinone oxidoreductase (*nuoC-N*), 7 genes encoding F-type ATP synthase (*atp*), 4 genes encoding succinate dehydrogenase (*frdA-D*), and 2 genes encoding the cytochrome bd complex (*cydA*, *cydX*).

Fatty acid oxidation is another crucial pathway for cellular energy production. Among the 8 DEGs related to this pathway, 7 showed downregulated expression (Fig. 11d). Genes encoding key enzymes involved in fatty acid β-oxidation, such as acyl-CoA synthetase (*fadA*, *fadK*), acyl-CoA dehydrogenase (*fadE*), hydroxyacyl-CoA dehydrogenase (*fadB*), and acetyl-CoA acyltransferase (*fadI*), all showed significantly downregulated expression in response to juglone treatment.

##### Nucleic acid synthesis and degradation

**Fig. 12:**
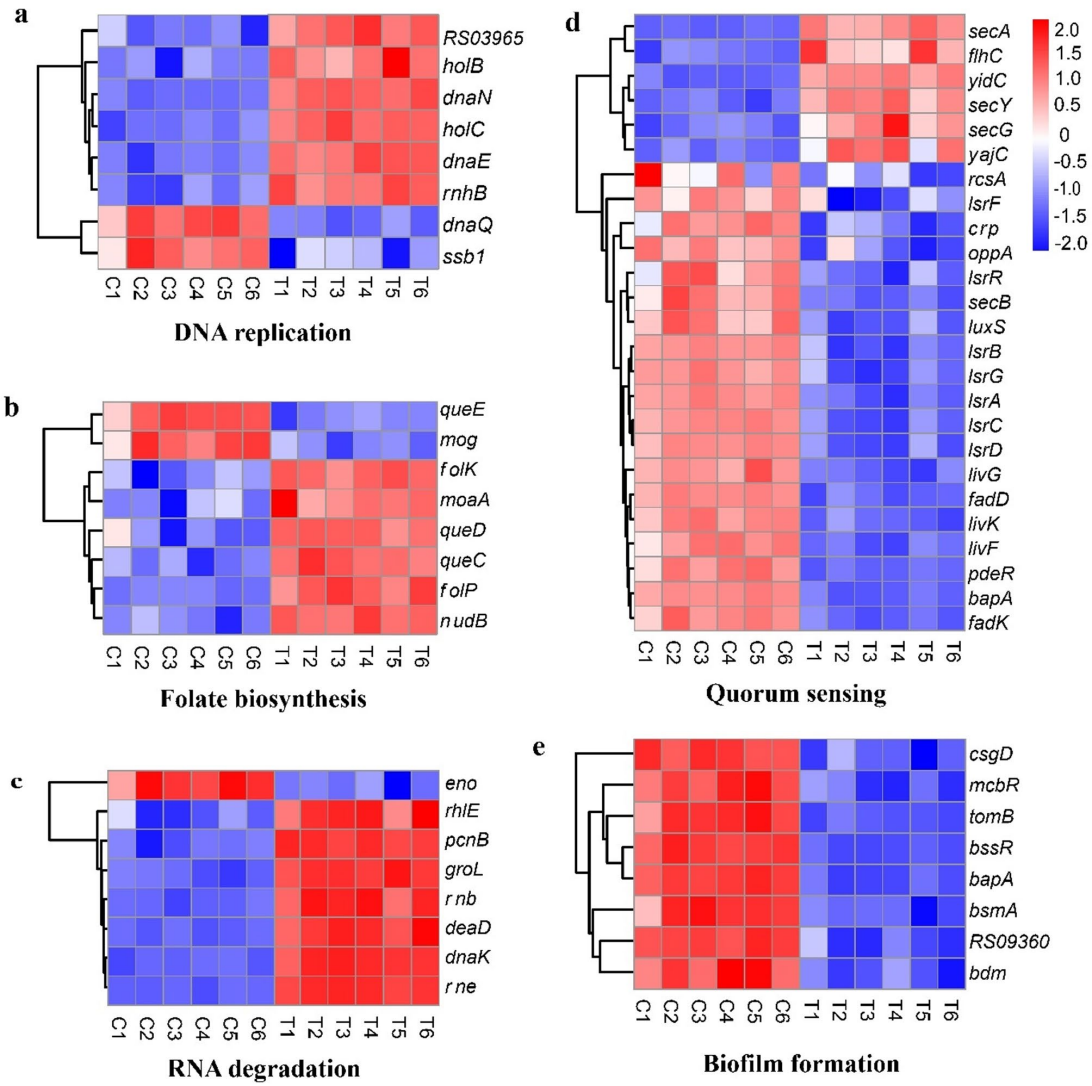
Heatmap of differentially expressed genes (DEGs) related to nucleic acid synthesis and degradation, and to quorum sensing and biofilms, ranked by gene set enrichment analysis (GSEA). (**a)** DNA replication. (**b)** Folate biosynthesis. (**c)** RNA degradation. (**d)** Quorum sensing. (**e)** Biofilm formation

Juglone had significant effects on DNA replication and RNA degradation, causing the expression of most DEGs to be upregulated. Among the DEGs related to DNA replication, those encoding DNA polymerase III (*dnaN*, *dnaE*, *holB*, *holC*, and *RS03965*) and RNase H (*rnhB*) were upregulated (Fig. 12a), whereas only genes encoding the DNA polymerase III ε subunit (*dnaQ*) and single-strand binding protein (*ssb1*) were downregulated.

A total of 8 DEGs were identified in the folate biosynthesis pathway, 6 of which were upregulated. Key enzymes involved in folate synthesis, such as dihydropteroate synthase (*folP*), 6-hydroxymethyl-7,8-dihydropterin pyrophosphokinase (*folK*), and 6-pyruvoyl tetrahydropterin synthase (*queD*), were upregulated (Fig. 12b).

In the RNA degradation pathway, genes encoding RNase E (*rne*), RNase II (*rnb*), RNA helicases (*deaD*, *rhlE*), and molecular chaperones (*dnaK*, *groL*) were upregulated (Fig. 12c).

##### Quorum sensing and biofilm formation

Juglone treatment led to the downregulation of expression of 19/25 DEGs related to the *S. pullorum* quorum sensing (QS) pathway (Fig. 12d). The downregulated genes included primarily those involved in fatty acid activation (*fadD*, *fadK*), branched-chain amino acid transport (*livF*, *livG*, and *livK*), oligopeptide transport (*oppA*), autoinducer 2 (AI-2) synthesis (*lsrA*, *lsrB*, *lsrC*, *lsrD*, *lsrF*, *lsrG*, and *lsrR*), S-ribosylhomocysteine lyase (*luxS*), and the protein-exporter chaperone (*SecB*). In contrast, genes related to protein translocation (*secA*, *secG*, *secY*, and *yajC*) and inner membrane proteins *(yidC*) were upregulated. Genes associated with biofilm formation were also downregulated (Fig. 12e). These genes included those encoding biofilm matrix proteins (*bapA*, *bdm*) and regulatory factors involved in biofilm formation (*bssR*, *tomB*, *csgD*, *RS09360*, and *mcbR*).

## Discussion

The cell wall and cell membrane are crucial barriers that protect cells from adverse external environments and play important roles in bacterial growth and proliferation. Gram-negative bacteria have relatively thin but structurally complex cell walls, typically consisting of an outer membrane, a peptidoglycan layer, and a periplasmic space. The outer membrane is composed of a lipoprotein bilayer and lipopolysaccharides [26]. Additionally, porins are embedded in the outer membrane to facilitate the transport of small molecules, such as iron ions. The periplasm is a multifunctional compartment located between the outer membrane and the inner membrane. It contains structural components; environmental sensing modules, including periplasmic binding proteins; and enzymes involved in macromolecule degradation and detoxification [27, 28]. The inner membrane, also known as the cytoplasmic membrane, is primarily composed of a phospholipid bilayer and numerous membrane proteins. These proteins are crucial for various cellular functions, including material transport, energy transduction, and signal transduction [29]. When the integrity of the cell membrane and cell wall is disrupted, ions and biomolecules (nucleic acids and proteins) leak into the culture media, and cellular functions are impaired, leading to cell death in severe cases.

Previous studies have found that juglone increases cell membrane permeability and disrupts membrane integrity by binding to bacterial cell membranes, inducing the formation of pores, vesicles, and tubules, as well as impairing membrane potential. This results in the leakage of intracellular proteins, nucleic acids, ATP, and other cellular components [23, 30, 31]. In this study, juglone treatment increased culture media conductivity and extracellular AKP activity and decreased the DNA and RNA fluorescence intensity, indicating disrupted cell membrane permeability. Juglone may disrupt the structure and function of bacterial membranes through three primary mechanisms. First, it interacts with bacterial membrane proteins, altering their conformation and thereby impairing normal membrane function. For instance, in *E. coli*, juglone binds to membrane proteins, leading to structural disorganization of the membrane [32]. Second, juglone decreases antioxidant enzyme activity, increases malondialdehyde content, generates ROS, and induces oxidative stress, which subsequently causes lipid peroxidation in the membrane [20, 23, 33]. This oxidative stress may be associated with excessive oxidative phosphorylation. During redox reactions in the electron transport chain complexes, ROS are produced, which can cause cell membrane damage and cellular senescence [34]. Third, it disrupts the balanced expression of genes involved in cell membrane synthesis, further compromising membrane integrity. In this study, the upregulation of genes involved in lipopolysaccharide and peptidoglycan synthesis suggests that juglone damages the cell wall and membrane structures, prompting the cell to accelerate the synthesis of essential components to repair the extracellular structure. Concurrently, the downregulation of amino acid transporter-encoding genes reduces enzyme and lipoprotein synthesis, subsequently inhibiting cell viability. Additionally, the downregulation of genes encoding outer membrane lipoproteins exacerbates membrane damage. FESEM confirmed these findings by revealing depressions and ruptures on the bacterial surfaces.

Based on the data from this study, juglone severely disrupts the energy metabolism of *S. pullorum*. Oxidative phosphorylation is the primary pathway for ATP synthesis and is a critical component of bacterial energy metabolism. ATP drives bacterial biosynthesis, motility, and quorum sensing. After treatment with walnut green husk extract, *E. coli* upregulates key enzyme-encoding genes involved in glycolysis, the TCA cycle, and oxidative phosphorylation, while downregulating those in the gluconeogenesis pathway [14]. In the DEGs identified in this study, key genes associated with glycolysis and fatty acid oxidation exhibited downregulated expression levels, while most DEGs related to gluconeogenesis, the TCA cycle, and oxidative phosphorylation were upregulated. However, a significant decrease in intracellular ATP concentration was observed. This seemingly contradictory phenomenon may be attributed to the following mechanisms. Firstly, as previously discussed, juglone-induced structural damage to cellular membranes likely causes ATP and ATPase leakage, thereby disrupting normal energy metabolism and ultimately leading to cellular energy deficiency. Due to the leakage of ATP and ATPase, cells compensate for the upregulation of the TCA cycle and oxidative phosphorylation pathways in an attempt to maintain cellular energy homeostasis. Secondly, juglone may trigger a metabolic reprogramming strategy in bacteria to cope with stress, remodeling key pathways involved in glycolysis, the Krebs cycle, oxidative phosphorylation, lipid biosynthesis, and amino acid metabolism to optimize bioenergetic resources and ensure survival under hostile conditions [35]. Thirdly, juglone may induce uncoupling of oxidative phosphorylation, dissipating the proton motive force as heat rather than driving ATP synthesis. This results in an “inefficient cycle” between the Krebs cycle and oxidative phosphorylation, where substrate oxidation continues without net ATP production. Although no direct evidence currently demonstrates juglone’s involvement in uncoupling bacterial oxidative phosphorylation, study has shown that it acts as a mitochondrial uncoupler in rat hepatocytes. In this context, juglone stimulates substrate oxidation while paradoxically reducing ATP levels and causing ADP/AMP accumulation [36]. Notably, high doses of juglone may pose risks similar to those associated with classical uncouplers like 2,4-dinitrophenol (DNP), including severe impairment of ATP synthesis, hyperthermia, and even lethality [36]. Therefore, excessive oxidative phosphorylation can further exacerbate the cellular burden and premature cellular aging, ultimately resulting in cell death [34]. Although juglone is clearly established as a respiratory chain inhibitor in eukaryotic cells, further experiments are necessary to directly confirm its role in bacterial ATP synthesis, electron transport, and particularly its potential uncoupling effect on oxidative phosphorylation.

The DNA damage induced by juglone in *S. pullorum* may occur through multiple mechanisms. On one hand, juglone-generated ROS not only directly damage DNA but also delay the repair of DNA lesions, ultimately leading to chromosomal aberrations [33, 37]. On the other hand, the naphthoquinone plane of juglone embeds in the region between two TA base pairs within the DNA groove, distorting the helical conformation of the DNA and consequently interfering with transcriptional activity and the replication process [30, 38]. In this study, the majority of DEGs associated with ribosomes, DNA replication, RNA degradation, and folate biosynthesis were upregulated. Similar findings have been reported previously by Wang et al. [14]. Notably, juglone treatment disrupts extracellular structures, leading to the leakage of nucleic acids and proteins. In response to this disturbance, cells attempt to maintain homeostasis by increasing DNA replication, transcription, and translation. Hu et al. [39] reported that *Perilla frutescens* essential oil (PEO) induces the upregulation of RNA transcription- and translation-related enzymes in *Aspergillus flavus*, which may represent a protective response to the PEO-induced reduction in protein content. Folate, a one-carbon donor and coenzyme in nucleic acid synthesis, plays a role in the biosynthesis of DNA and RNA and is involved in the methylation of ribosomal RNA and DNA, thus influencing gene expression [40]. The upregulation of key enzyme-encoding genes involved in folate synthesis is closely related to increased levels of DNA replication and transcription.

Bacterial biofilms represent a protective survival state by which bacteria adapt to their surrounding environment during growth. Compared with planktonic bacteria, bacteria within biofilms exhibit increased resistance to environmental stresses such as antimicrobial agents, desiccation, and attack by a host defense system [41]. QS is closely related to biofilm formation, thereby activating the maturation and dispersal of biofilms in a coordinated manner [41]. AI-2, synthesized by LuxS, is a ubiquitous QS-related signaling molecule found in both gram-negative and gram-positive bacteria that plays a crucial role in regulating biofilm development [42, 43]. In this study, the downregulation of genes encoding AI-2 and LuxS indicates that juglone treatment diminishes QS-related signal transduction by inhibiting the LuxS/AI-2 system, thereby reducing biofilm formation. The biofilm matrix, which is composed of exopolysaccharides, amyloidogenic proteins, extracellular DNA, and lipids, plays a significant role in adhesion and digestion [44]. Research has shown that inhibition of the LuxS/AI-2 QS system in *E. coli* alters the expression of genes related to flagellar assembly, chemotaxis, and adhesion factors, thereby suppressing biofilm formation and modifying bacterial community adhesion states [45]. Additionally, juglone also inhibits biofilm formation by reducing the metabolic activity of bacterial communities, restricting bacterial motility, and causing membrane depolarization [31, 46]. In addition to regulating biofilm formation, QS also controls bacterial toxin secretion and bacteriocin synthesis [47, 48]. In this study, juglone treatment led to a decrease in the expression levels of genes related to fatty acid activation and the transportation of branched-chain amino acids, oligopeptides and proteins, indicating that the inhibition of QS in *S. pullorum* disrupts interpopulation communication and reduces the ability to withstand adverse environments.

### Limitations

While this work elucidates juglone’s antibacterial mechanisms via transcriptomics, certain limitations should be noted. First, our study focused on in vitro antibacterial assays and transcriptomic analysis. While these results provide mechanistic insights, further in *vivo* experiments are necessary to confirm juglone’s efficacy and safety in a physiological context. Second, Juglone’s potential cytotoxicity to host cells and its environmental impacts were not evaluated in this study. Future research should assess its toxicity to mammalian cells to ensure therapeutic applicability. Finally, the study primarily examined *S. pullorum*; expanding the research to include other clinically relevant strains would strengthen the generalizability of the findings.

## Conclusion

Our study demonstrates that juglone compromises cell membrane integrity and increases membrane permeability in *E. coli*, *S. aureus*, and *S. pullorum*, resulting in cytoplasmic leakage and reduced metabolic activity. Transcriptomic analysis of *S. pullorum* further revealed that juglone alters the gene expression of transporters, interferes with pathways related in energy metabolism, protein synthesis, and quorum sensing, and biofilm formation at the transcriptional level in this species. While membrane disruption appears to be a conserved antibacterial mechanism across all three bacteria, the downstream transcriptional responses observed in *S. pullorum* require further validation in *E. coli* and *S. aureus* to determine their broader applicability. These findings collectively highlight juglone’s potential as a multitarget antimicrobial agent, though species-specific mechanistic differences may exist.

## Supplementary Information

Supplementary Material 1.

## Data Availability

The RNA-seq data were deposited in NCBI data repository are available at the following URL: https://www.ncbi.nlm.nih.gov/sra/PRJNA1167005. All the other data generated and analyzed has already been incorporated into the study.

## Abbreviations

ROS: Reactive oxygen species
AKP: Alkaline phosphatase
MIC: Minimum inhibitory concentration
DAPI: 4’,6-Diamidino-2-phenylindole
INT: Iodonitrotetrazolium chloride
CFU: Colony-forming units
FFA: Formalin-aceto-alcohol fixative
FESEM: Field emission scanning electron microscope
FPKMs: Fragments per kilobase of transcript per million
GO: Gene Ontology
KEGG: Kyoto Encyclopedia of Genes and Genomes
DEGs: Differentially expressed genes
PCA: Principal component analysis
BPs: Biological processes
CCs: Cellular components
MFs: Molecular functions
ABC: ATP-binding cassette
LPS: Lipopolysaccharide
PG: Peptidoglycan
TCA: Tricarboxylic acid
QS: Quorum sensing
AI-2: Autoinducer 2

## References

[1] Wang K, Cha J, Liu K, Deng J, Yang B, Xu H, Wang J, Zhang L, Gu X, Huang C, Qu W. The prevalence of bovine mastitis-associated *Staphylococcus aureus* in China and its antimicrobial resistance rate: A meta-analysis. Front Vet Sci. 2022;9:1006676. 10.3389/fvets.2022.1006676. 36439336 10.3389/fvets.2022.1006676 PMC9687384

[2] Wibisono FJ, Sumiarto B, Kusumastuti TA. Economic losses Estimation of pathogenic *Escherichia coli* infection in Indonesian poultry farming. Bull Anim Sci. 2018;42(4):341–6. 10.21059/buletinpeternak.v42i4.37505.

[3] Chen Y, Zhu F, Yu G, Peng N, Li X, Ge M, Yang L, Dong W. *Bifidobacterium bifidum* postbiotics prevent *Salmonella pullorum* infection in chickens by modulating pyroptosis and enhancing gut health. Poult Sci. 2025;104(4):104968. 10.1016/j.psj.2025.104968. 40043668 10.1016/j.psj.2025.104968 PMC11927735

[4] Antimicrobial Resistance Collaborators. Global burden of bacterial antimicrobial resistance in 2019: A systematic analysis. Lancet. 2022;399(10325):629–55. 10.1016/S0140-6736(21)02724-0. 35065702 10.1016/S0140-6736(21)02724-0 PMC8841637

[5] Pulingam T, Parumasivam T, Gazzali AM, Sulaiman AM, Chee JY, Lakshmanan M, Chin CF, Sudesh K. Antimicrobial resistance: prevalence, economic burden, mechanisms of resistance and strategies to overcome. Eur J Pharm Sci. 2022;170:106103. 10.1016/j.ejps.2021.106103. 34936936 10.1016/j.ejps.2021.106103

[6] Anand Kumar P. Antimicrobial resistance in animal sector. In: Mothadaka MP, Vaiyapuri M, Rao Badireddy M, Nagarajrao Ravishankar C, Bhatia R, Jena J, editors. Handbook on antimicrobial resistance. Singapore: Springer; 2023. pp. 21–37. 10.1007/978-981-19-9279-7_4.

[7] Abreu R, Semedo-Lemsaddek T, Cunha E, Tavares L, Oliveira M. Antimicrobial drug resistance in poultry production: current status and innovative strategies for bacterial control. Microorganisms. 2023;11(4):953. 10.3390/microorganisms11040953. 37110376 10.3390/microorganisms11040953 PMC10141167

[8] Kikusato M. Phytobiotics to improve health and production of broiler chickens: functions beyond the antioxidant activity. Anim Biosci. 2021;34(3):345–53. 10.5713/ab.20.0842. 33705621 10.5713/ab.20.0842 PMC7961201

[9] Elbaz AM, Ashmawy ES, Ali SAM, Mourad DM, El-Samahy HS, Badri FB, Thabet HA. Effectiveness of probiotics and clove essential oils in improving growth performance, immuno-antioxidant status, ileum morphometric, and microbial community structure for heat-stressed broilers. Sci Rep. 2023;13(1):18846. 10.1038/s41598-023-45868-9. 37914748 10.1038/s41598-023-45868-9 PMC10620235

[10] Ibrahim D, Eldemery F, Metwally AS, Abd-Allah EM, Mohamed DT, Ismail TA, Hamed TA, Al Sadik GM, Neamat-Allah ANF, Abd El-Hamid MI. Dietary Eugenol nanoemulsion potentiated performance of broiler chickens: orchestration of digestive enzymes, intestinal barrier functions and cytokines related gene expression with a consequence of attenuating the severity of *E. coli* O78 infection. Front Vet Sci. 2022;9:847580. 10.3389/fvets.2022.847580. 35812892 10.3389/fvets.2022.847580 PMC9260043

[11] Aljuwayd M, Malli IA, Kwon YM. Application of Eugenol in poultry to control Salmonella colonization and spread. Vet Sci. 2023;10(2):151. 10.3390/vetsci10020151. 36851455 10.3390/vetsci10020151 PMC9962070

[12] Soliman MM, Elsaba YM, Soliman MSA, Ahmed EZ. Composition and antimicrobial activity of *Rosmarinus officinalis* L. and *Artemisia monosperma* L. leaf essential oils and methanolic extracts from plants grown in normal and saline habitats in Egypt. Sci Rep. 2024;14(1):7342. 10.1038/s41598-024-57301-w. 38538682 10.1038/s41598-024-57301-w PMC10973528

[13] Yao Y, Liu Y, Li C, Huang X, Zhang X, Deng P, Jiang G, Dai Q. Effects of Rosemary extract supplementation in feed on growth performance, meat quality, serum biochemistry, antioxidant capacity, and immune function of meat ducks. Poult Sci. 2023;102(2):102357. 10.1016/j.psj.2022.102357. 36502565 10.1016/j.psj.2022.102357 PMC9763849

[14] Wang L, Li W, Li X, Liu J, Chen Y. Antimicrobial activity and mechanisms of walnut green husk extract. Molecules. 2023;28(24):7981. 10.3390/molecules28247981. 38138470 10.3390/molecules28247981 PMC10745604

[15] Wan Y, Wang X, Yang L, Li Q, Zheng X, Bai T, Wang X. Antibacterial activity of juglone revealed in a wound model of *Staphylococcus aureus* infection. Int J Mol Sci. 2023;24(4):3931. 10.3390/ijms24043931. 36835350 10.3390/ijms24043931 PMC9963570

[16] Cai Y, Zou G, Xi M, Hou Y, Shen H, Ao J, Li M, Wang J, Luo A. Juglone inhibits *Listeria monocytogenes* ATCC 19115 by targeting cell membrane and protein. Foods. 2022;11(17):2558. 10.3390/foods11172558. 36076744 10.3390/foods11172558 PMC9455723

[17] Arasoglu T, Derman S, Mansuroglu B, Yelkenci G, Kocyigit B, Gumus B, Acar T, Kocacaliskan I. Synthesis, characterization and antibacterial activity of juglone encapsulated PLGA nanoparticles. J Appl Microbiol. 2017;123(6):1407–19. 10.1111/jam.13601. 28980369 10.1111/jam.13601

[18] Majdi C, Duvauchelle V, Meffre P, Benfodda Z. An overview on the antibacterial properties of juglone, naphthazarin, Plumbagin and Lawsone derivatives and their metal complexes. Biomed Pharmacother. 2023;162:114690. 10.1016/j.biopha.2023.114690. 37075666 10.1016/j.biopha.2023.114690

[19] Liu Z, Shen Z, Xiang S, Sun Y, Cui J, Jia J. Evaluation of 1,4-naphthoquinone derivatives as antibacterial agents: activity and mechanistic studies. Front Environ Sci Eng. 2023;17(3):31. 10.1007/s11783-023-1631-2. 36313056 10.1007/s11783-023-1631-2 PMC9589524

[20] Ahmad T, Suzuki YJ. Juglone in oxidative stress and cell signaling. Antioxid (Basel). 2019;8(4):91. 10.3390/antiox8040091. 10.3390/antiox8040091 PMC6523217 30959841

[21] Vardhini SR. Exploring the antiviral activity of juglone by computational method. J Recept Signal Transduct Res. 2014;34(6):456–7. 10.3109/10799893.2014.917325. 24846583 10.3109/10799893.2014.917325

[22] Wang J, Wang Z, Wu R, Jiang D, Bai B, Tan D, Yan T, Sun X, Zhang Q, Wu Z. Proteomic analysis of the antibacterial mechanism of action of juglone against *Staphylococcus aureus*. Nat Prod Commun. 2016a;11(6):825–7. 10.1177/1934578X1601100632. 27534127

[23] Wang J, Cheng Y, Wu R, Jiang D, Bai B, Tan D, Yan T, Sun X, Zhang Q, Wu Z. Antibacterial activity of juglone against *Staphylococcus aureus*: from apparent to proteomic. Int J Mol Sci. 2016b;17(6):965. 10.3390/ijms17060965. 27322260 10.3390/ijms17060965 PMC4926497

[24] Zgoda JR, Porter JR. A convenient microdilution method for screening natural products against bacteria and fungi. Pharm Biol. 2001;39:221–5. 10.1076/phbi.39.3.221.5934.

[25] Bradford MM. A rapid and sensitive method for the quantitation of microgram quantities of protein utilizing the principle of protein-dye binding. Anal Biochem. 1976;72:248–54. 10.1016/0003-2697(76)90527-3. 942051 10.1016/0003-2697(76)90527-3

[26] Beveridge TJ. Structures of gram-negative cell walls and their derived membrane vesicles. J Bacteriol. 1999;181(16):4725–33. 10.1128/JB.181.16.4725-4733.1999. 10438737 10.1128/jb.181.16.4725-4733.1999 PMC93954

[27] Miller SI, Salama NR. The gram-negative bacterial periplasm: size matters. PLoS Biol. 2018;16(1):e2004935. 10.1371/journal.pbio.2004935. 29342145 10.1371/journal.pbio.2004935 PMC5771553

[28] Edwards KA. Periplasmic-binding protein-based biosensors and bioanalytical assay platforms: advances, considerations, and strategies for optimal utility. Talanta Open. 2021;3:100038. 10.1016/j.talo.2021.100038.

[29] Facey SJ, Kuhn A. Biogenesis of bacterial inner-membrane proteins. Cell Mol Life Sci. 2010;67(14):2343–62. 10.1007/s00018-010-0303-0. 20204450 10.1007/s00018-010-0303-0 PMC11115511

[30] Han Q, Feng L, Zhang Y, Zhang R, Wang G, Zhang Y. Effect of juglone against *Pseudomonas syringae pv actinidiae* planktonic growth and biofilm formation. Molecules. 2021a;26(24):7580. 10.3390/molecules26247580. 34946659 10.3390/molecules26247580 PMC8705749

[31] Han Q, Yan X, Zhang R, Wang G, Zhang Y. Juglone inactivates *Pseudomonas aeruginosa* through cell membrane damage, biofilm blockage, and Inhibition of gene expression. Molecules. 2021b;26(19):5854. 10.3390/molecules26195854. 34641398 10.3390/molecules26195854 PMC8510502

[32] Lu Z, Wu Q, Zhang J, Mao X. Antibacterial effect and mechanism of juglone from walnut green husk against *Escherichia coli*. Food Sci. 2023;44(7):65–73. 10.7506/spkx1002-6630-20220318-215.

[33] Altuntaş H, Duman E, Kılıç G. Juglone induced oxidative and genotoxic stress in the model insect *Galleria Mellonella* L. (Pyralidae: Lepidoptera). Int J Trop Insect Sci. 2020;40:611–9. 10.1007/s42690-020-00107-w.

[34] Dogovski C, Xie SC, Burgio G, Bridgford J, Mok S, McCaw JM, Chotivanich K, Kenny S, Gnädig N, Straimer J, Bozdech Z, Fidock DA, Simpson JA, Dondorp AM, Foote S, Klonis N, Tilley L. Targeting the cell stress response of *Plasmodium falciparum* to overcome Artemisinin resistance. PLoS Biol. 2015;13:e1002132. 10.1371/journal.pbio.1002132. 25901609 10.1371/journal.pbio.1002132 PMC4406523

[35] Acierno C, Barletta F, Nevola R, Rinaldi L, Sasso FC, Adinolfi LE, Caturano A. Metabolic rewiring of bacterial pathogens in response to antibiotic pressure—A molecular perspective. Int J Mol Sci. 2025;26(12):5574. 10.3390/ijms26125574. 40565037 10.3390/ijms26125574 PMC12193369

[36] Saling SC, Comar JF, Mito MS, Peralta RM, Bracht A. Actions of juglone on energy metabolism in the rat liver. Toxicol Appl Pharmacol. 2011;257(3):319–27. 10.1016/j.taap.2011.09.004. 21945490 10.1016/j.taap.2011.09.004

[37] Tang YT, Li Y, Chu P, Ma XD, Tang ZY, Sun ZL. Molecular biological mechanism of action in cancer therapies: juglone and its derivatives, the future of development. Biomed Pharmacother. 2022;148:112785. 10.1016/j.biopha.2022.112785. 35272138 10.1016/j.biopha.2022.112785

[38] Shen B, Yang H, Chen J, Liu X, Zhou M. Study the interaction between juglone and calf thymus DNA by spectroscopic and molecular Docking techniques. Spectrochim Acta Mol Biomol Spectrosc. 2021;261:119998. 10.1016/j.saa.2021.119998. 10.1016/j.saa.2021.119998 34091358

[39] Hu Z, Lu C, Zhang Y, Tong W, Du L, Liu F. Proteomic analysis of *Aspergillus flavus* reveals the antifungal action of *Perilla frutescens* essential oil by interfering with energy metabolism and defense function. LWT. 2022;154:112660. 10.1016/j.lwt.2021.112660.

[40] Stover PJ. One-carbon metabolism-genome interactions in folate-associated pathologies. J Nutr. 2009;139(12):2402–5. 10.3945/jn.109.113670. 19812215 10.3945/jn.109.113670 PMC2777484

[41] Solano C, Echeverz M, Lasa I. Biofilm dispersion and quorum sensing. Curr Opin Microbiol. 2014;18:96–104. 10.1016/j.mib.2014.02.008. 24657330 10.1016/j.mib.2014.02.008

[42] Song XD, Liu CJ, Huang SH, Li XR, Yang E, Luo YY. Cloning, expression and characterization of two S-ribosylhomocysteine lyases from *Lactobacillus plantarum* YM-4-3: implication of conserved and divergent roles in quorum sensing. Protein Expr Purif. 2018;145:32–8. 10.1016/j.pep.2017.12.013. 29305177 10.1016/j.pep.2017.12.013

[43] Wang Y, Bian Z, Wang Y. Biofilm formation and Inhibition mediated by bacterial quorum sensing. Appl Microbiol Biotechnol. 2022;106(19–20):6365–81. 10.1007/s00253-022-12150-3. 36089638 10.1007/s00253-022-12150-3

[44] Behura A, Das M, Kumar A, Naik L, Patel S, Nayak DK, Mishra A, Mishra A, Dhiman R. Mycobacterial biofilm: structure and its functional relevance in the pathogenesis. In: Das S, Kungwani NA, editors. Understanding microbial biofilms, fundamentals to applications. San Diego: Academic; 2023. pp. 461–74. 10.1016/B978-0-323-99977-9.00018-1.

[45] Zhang C, Li C, Aziz T, Alharbi M, Cui H, Lin L. Screening of *E. coli* O157:H7 AI-2 QS inhibitors and their inhibitory effect on biofilm formation in combination with disinfectants. Food Biosci. 2024;58:103821. 10.1016/j.fbio.2024.103821.

[46] Gumus B, Acar T, Atabey T, Derman S, Sahin F, Arasoglu T. The battle against biofilm infections: juglone loaded nanoparticles as an anticandidal agent. J Biotechnol. 2020;316:17–26. 10.1016/j.jbiotec.2020.04.009. 32315688 10.1016/j.jbiotec.2020.04.009

[47] García-Curiel L, Del Rocío López-Cuellar M, Rodríguez-Hernández AI, Chavarría-Hernández N. Toward Understanding the signals of bacteriocin production by *Streptococcus* spp. And their importance in current applications. World J Microbiol Biotechnol. 2021;37(1):15. 10.1007/s11274-020-02973-5. 33394178 10.1007/s11274-020-02973-5

[48] Tripathi S, Purchase D, Govarthanan M, Chandra R, Yadav S. Regulatory and innovative mechanisms of bacterial quorum sensing-mediated pathogenicity: A review. Environ Monit Assess. 2022;195(1):75. 10.1007/s10661-022-10564-0. 36334179 10.1007/s10661-022-10564-0

