# Supplementary material for: Antimicrobial activity and possible mechanisms of juglone against Escherichia coli, Staphylococcus aureus, and Salmonella pullorum
Source: BMC Microbiol. 2025 Oct 17;25:668. doi: 10.1186/s12866-025-04354-0 (PMC12534947; doi:10.1186/s12866-025-04354-0)
Supplement: Supplementary file 1 — Supplementary Material 1. [file 12866_2025_4354_MOESM1_ESM.docx]

Table S1 Data quality

| Samples | Raw reads | Clean reads | Clean bases | Error rate (%) | Q20 | Q30 | GC (%) | Total mapping rate (%) | Uniquely mapping rate (%) | Multiple mapping rate (%) |
| --- | --- | --- | --- | --- | --- | --- | --- | --- | --- | --- |
| C1 | 15438434 | 15050090 | 2.3G | 0.02 | 98.14 | 94.68 | 53.34 | 98.77 | 92.40 | 6.37 |
| C2 | 15129686 | 14437006 | 2.2G | 0.02 | 98.28 | 95.01 | 53.27 | 98.75 | 97.42 | 1.33 |
| C3 | 16746390 | 16415608 | 2.5G | 0.02 | 98.32 | 95.11 | 53.25 | 98.82 | 95.24 | 3.58 |
| C4 | 15655184 | 14660364 | 2.2G | 0.02 | 98.23 | 94.90 | 53.29 | 98.90 | 94.39 | 4.51 |
| C5 | 16365404 | 15256352 | 2.3G | 0.02 | 98.06 | 94.54 | 53.19 | 98.77 | 97.18 | 1.59 |
| C6 | 14269412 | 13261974 | 2.0G | 0.02 | 98.19 | 94.83 | 53.28 | 98.81 | 96.24 | 2.57 |
| T1 | 14603366 | 13965172 | 2.1G | 0.02 | 98.15 | 94.74 | 54.46 | 98.67 | 96.23 | 2.44 |
| T2 | 17458400 | 16850714 | 2.5G | 0.02 | 98.06 | 94.52 | 53.50 | 98.76 | 95.04 | 3.72 |
| T3 | 15677340 | 15164922 | 2.3G | 0.02 | 98.07 | 94.51 | 53.79 | 98.78 | 94.41 | 4.37 |
| T4 | 15179736 | 14089576 | 2.1G | 0.02 | 98.22 | 94.91 | 53.81 | 98.81 | 93.24 | 5.58 |
| T5 | 15889548 | 15165498 | 2.3G | 0.02 | 98.08 | 94.57 | 54.66 | 98.75 | 96.55 | 2.19 |
| T6 | 15894870 | 14667764 | 2.2G | 0.02 | 98.24 | 94.94 | 53.95 | 98.76 | 93.18 | 5.59 |
